# A human 3D culture-organ-on-chip platform for investigating the tumor microenvironment response to ionizing radiation

**DOI:** 10.1016/j.isci.2025.114236

**Published:** 2025-11-26

**Authors:** Jerome Lacombe, Sean E. Dunn, Marie Layac, Maria Soldevila, Nabhan M. Fakrudin, Brett Duane, Kurt Chen, Matthew W. Barrett, James Helton, Evagelia C. Laiakis, Albert J. Fornace, Shyam Jani, Stephen Sorensen, Shunjiro Funaki, Aidnag Diaz, Frederic Zenhausern

**Affiliations:** 1 Center for Applied NanoBioscience and Medicine, College of Medicine Phoenix, University of Arizona, 475 North 5th Street, Phoenix, AZ 85004, USA; 2 Department of Basic Medical Sciences, College of Medicine Phoenix, University of Arizona, 425 N 5th St., Phoenix, AZ 85004, USA; 3 School of Pharmaceutical Sciences, University of Geneva, 1211 Geneva, Switzerland; 4 Department of Radiation Medicine, Georgetown University Medical Center, 3970 Reservoir Rd NW, Washington DC 20057, USA; 5 Department of Oncology, Georgetown University Medical Center, 3970 Reservoir Rd NW, Washington DC 20057, USA; 6 Department of Biochemistry and Molecular & Cellular Biology, Georgetown University Medical Center, 3970 Reservoir Rd NW, Washington DC 20057, USA; 7 Center for Metabolomic Studies, Georgetown University, Washington DC 20057, USA; 8 Dignity Health Cancer Institute at St. Joseph’s Hospital and Medical Center, 625 N 6th St, Phoenix, AZ 85004, USA; 9 Mitsubishi Gas Chemical Company, Inc., Tokyo, Japan; 10 Department of Biomedical Engineering, College of Engineering, The University of Arizona, Tucson, AZ 85721, USA; 11 HonorHealth Research Institute, Scottsdale, AZ 85258, USA

**Keywords:** Immunology, Biotechnology, Cell biology, Cancer systems biology

## Abstract

Studying complex human responses, such as radiation-induced effects in the tumor microenvironment (TME), requires advanced *in vitro* systems. Here, we present the Apparatus to Simulate Tumor and Reproduce Organs in an Interactive and Dynamic System (ASTEROIDS), which integrates three-dimensional cell culture with organ-on-chip technology. To characterize the ASTEROIDS, a lung TME was reproduced by co-culturing cancer spheroids with endothelial and fibroblast cells mimicking vascular and stromal compartments. The ASTEROIDS maintained cell viability, endothelial barrier formation, and spheroid zonal structure for seven days. Transcriptomic profiling revealed endothelial-tumor crosstalk, while perfused immune cells exhibited recruitment and immunoregulatory activation. Upon single fraction stereotactic irradiation, ASTEROIDS showed dose-dependent DNA damage, nuclear hypertrophy, endothelial barrier disruption, spheroid growth inhibition, and metabolomic alterations. Together, these results demonstrate that ASTEROIDS faithfully reproduces TME-level organization and responses, establishing its feasibility as a pre-clinical human model for studying radiation effects and tumor-immune interactions.

## Highlights

• ASTEROIDS combines 3D cell culture and organ-on-chip technology
• ASTEROIDS supports long-term cell viability and tissue barrier integrity
• ASTEROIDS enables cell-cell communication and tumor-immune crosstalk
• ASTEROIDS provides a human-relevant platform for radiobiology and therapy testing

## Graphical abstract

**Figure:**
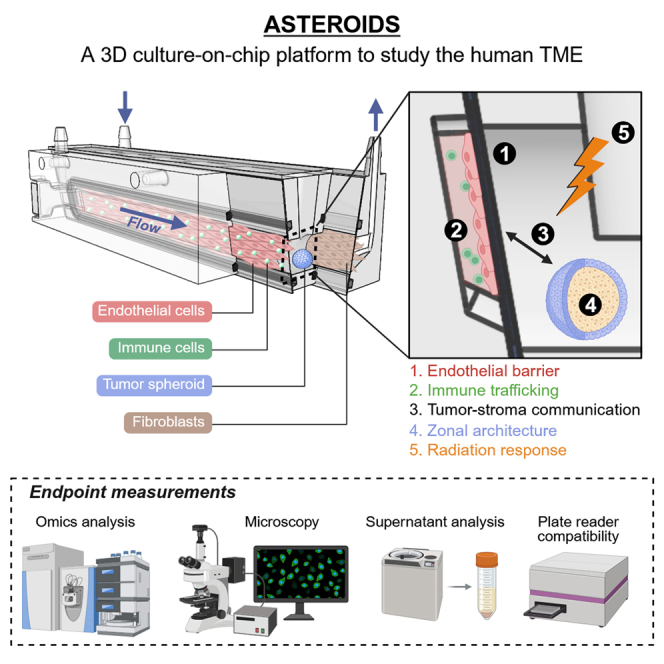

## Introduction

Major advances in fundamental, translational, and clinical oncology research rely on the need for non-animal models that can recapitulate the complex human tumor microenvironment (TME) more accurately. This TME provides a unique biological landscape, including multiple properties through its three-dimensional (3D) cellular morphology, the biochemical signaling governing the interaction of its multi-cellular components, and the mechanical forces occurring during tumor initiation, progression, invasion, and dissemination.^1^ Such complexity is essential not only for understanding basic tumor biology, but also for studying multifaceted mechanisms such as radiation-induced responses. These responses involve intricate processes such as DNA damage repair, immune system activation, bystander effects, and dynamic stromal-immune crosstalk that collectively shape therapeutic outcomes.^2^^,^^3^ Recently, improvement of biological models has led to the emergence of two new approaches: (1) the culture of 3D cellular structures (i.e., spheroids and organoids)^4^ and (2) the use of microfluidic devices reproducing a specific tissue function also known as organ-on-chip (OoC).^5^^,^^6^ First, 3D structures allow cells to establish native-like cell-cell and cell-matrix interactions and generate physiological gradients of oxygen, nutrients, and signals. This creates heterogeneous populations, including proliferating, quiescent, and necrotic cells, and a spatial organization closer to *in vivo* tissues. In the case of organoids, these structures also possess self-organizing and self-assembling capacities, enabling differentiation into distinct lineages that recapitulate organ-specific structure and function.^7^ Nevertheless, such structures are still limited by their static cell culture environment, implying uncontrolled delivery of compounds, non-physiological cell-to-media ratio, uncontrolled shear forces during media exchanges, as well as highly variable conditions between media exchanges.^8^ In addition, these structures are usually cultured alone and thus lack a cellular microenvironment and accurate organ representation.^9^ Alternatively, OoC devices contain small chambers for cell culture, enabling control over local gradients, fluid flow, tissue mechanics, and cellular composition of the local environment.^10^ Therefore, they allow the presence and integration of multiple cell types to reflect a more physiological balance of cells and presence of biomechanical forces relevant to tissues being modeled.^11^ However, even with these features, high cellular fidelity is usually not reproduced, mainly due to the lack of 3D structure. Moreover, OoC technologies are often created at the expense of throughput, industry-standard materials and form factors, and compatibility with state-of-the-art data collection tools.^12^ Therefore, in order to create a relevant *in vitro* model mimicking the human *in vivo* TME, there is a need to integrate 3D cell culture in OoC platforms through synergistic engineering.

To this end, we developed an Apparatus to Simulate Tumor Environment and Reproduce Organs using an Interactive and Dynamic System, referred to as ASTEROIDS. As a proof-of-concept to characterize the platform and demonstrate its capabilities, we aimed to reproduce a lung TME where lung cancer spheroids were cultured in close contact with multiple non-cancerous tissues in a physiologically relevant physical microenvironment. We showed that the ASTEROIDS allowed the mechanical and biochemical interactions between the different cells, thus recapitulating key tissue hallmarks previously described with *in vivo* observations and allowing the study of radiation response.

## Results

### Design and fluidics characterization of the ASTEROIDS model

**Figure 1:**
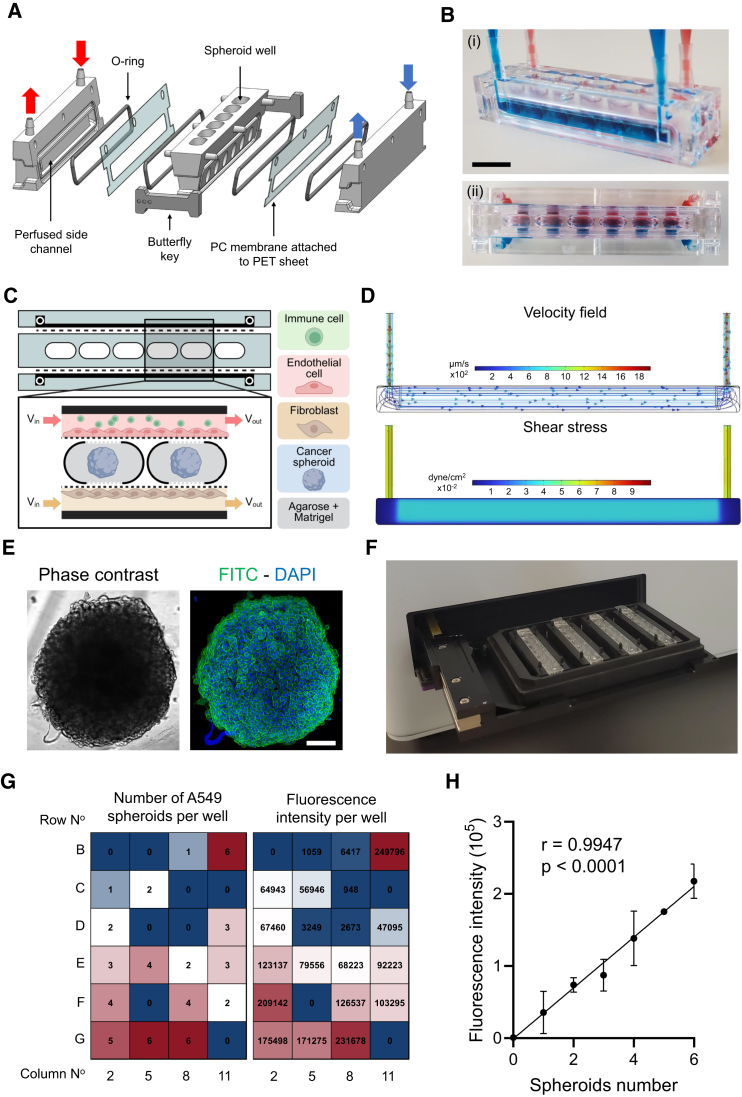
The ASTEROIDS model (A) Annotated exploded view of the ASTEROIDS device. (B) Photograph of the assembled ASTEROIDS device shown from the side (i) and top (ii) with blue and red dye-perfused water flowing through the side chambers. Scale bar = 1 cm. (C) Conceptual diagram of the ASTEROIDS model for the culture of tumor microenvironment. Created in BioRender. (D) Simulated velocity field and shear stress within the ASTEROIDS device at flow rate = 60 μL/min. (E) Bright field and fluorescence pictures of A549 spheroids stained with Phalloidin-FITC (green), DAPI (blue) imaged directly within the ASTEROIDS device. Scale bar = 100 μm. (F) ASTEROIDS devices loaded in a 96-well plate format carrier and inserted in a microplate reader. (G) Heatmaps represent the number of A549 spheroids loaded in each well of 4 ASTEROIDS devices placed in a 96-well plate format carrier (left) and the corresponding fluorescence intensity measured by microplate reader (right). (H) Spearman’s rank correlation between the number of spheroids and measured fluorescence intensity. *n* = 3. Data are represented as mean ± s.e.m.

The ASTEROIDS device consists of three main pieces: a central element with 6 round-bottom wells surrounded by two independent perfusion chambers on each side (Figures 1A–1C). The total volume of each well (V_well_ = 240 mm^3^) is sufficient to facilitate sustained growth of spherical tissue whose diameter is >500 μm.^13^ Each side chamber is separated from the central piece by a collagen-coated 8-μm pore size polycarbonate (PC) membrane. The membrane is attached to a polyester (PET) sheet and sealed between the chambers and the central piece with silicone O-rings mechanically clamped with two butterfly keys positioned on each extremity of the assembled device (Figure 1A). Such configuration allowed the culture of 3D cellular structures in the central wells, while non-cancerous cells can be seeded on the membrane to reproduce a physiological barrier and cellular and molecular exchanges at the tumor-normal interface (e.g., vascular and stromal compartments) (Figure 1C). Given the dimension of the device (Figure S1), the chambers can be perfused at a flow rate of 60 μL/min to achieve a flow velocity of 300 μm/s, similar to the average blood flow velocity measured in human tumor blood vessels.^14^^,^^15^ The resulting average shear stress is 0.02 dyne/cm^2^. Considering the kinematic viscosity of cell culture media at 37°C, similar to water, at this flow rate, the flow in the chamber is characterized by a Reynolds number of 0.5, suggesting the presence of a creeping motion where viscous forces dominate over the inertial forces. Computational fluid dynamics simulation confirmed that the velocity field and shear stress are uniform and around sub-millimeter-per-second and milliPascal along the chamber, respectively (Figure 1D).

In addition, the design of the ASTEROIDS has been developed to provide a friendly user interface compatible with most laboratory settings and equipment. The clamping system allows for an easy disassembly and intact recovery of the cells seeded on the membranes for subsequent analysis. The device, and in particular the central piece, is optically clear for a direct and real-time visualization of the 3D cellular structures under a microscope (Figure 1E). The dimension of the ASTEROIDS chip (i.e., height, length, and well spacing, Figure S1) also matches standard 96-well plates. This feature allowed several ASTEROIDS devices to be loaded on a 96-well plate format carrier (Figure 1F) that can then be used in a plate reader. Figure 1G shows the simultaneous reading of four ASTEROIDS seeded with different numbers of fluorescent spheroids. The data showed that fluorescence intensity significantly correlated with the number of spheroids (r = 0.9936, *p* < 0.0001), demonstrating the ability of the ASTEROIDS carrier to allow the detection and quantification of seeded spheroids using a plate reader (Figure 1H).

### Cell culture and establishment of tissue barrier in the ASTEROIDS

**Figure 2:**
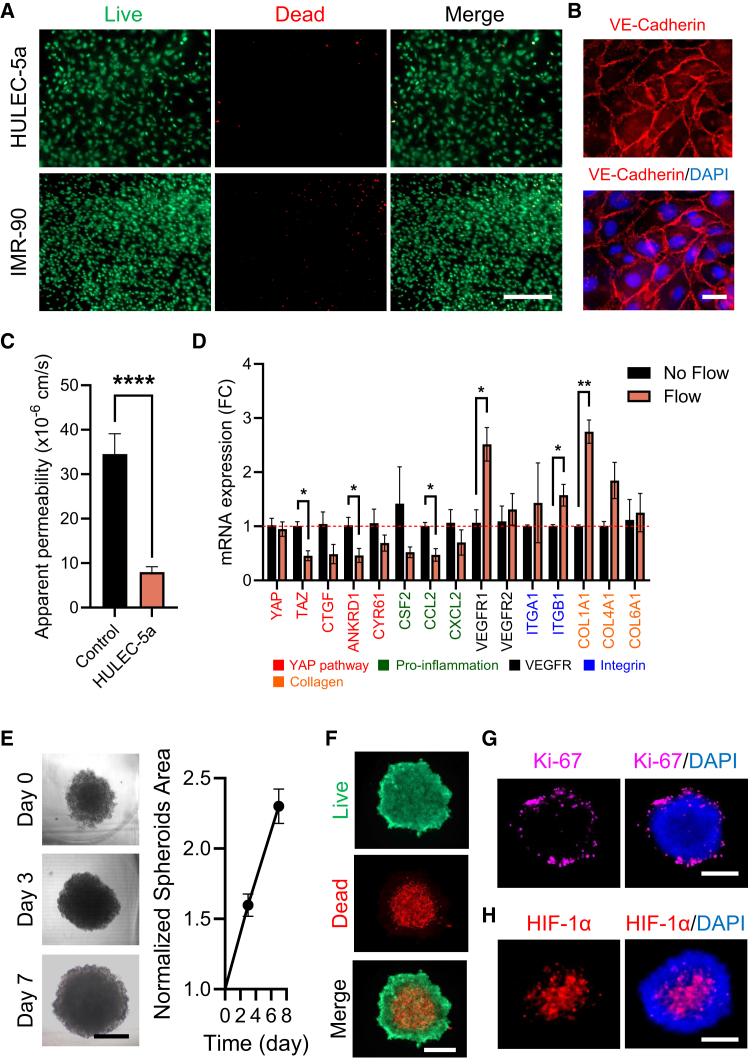
ASTEROIDS maintains cell viability and tissue morphology after 7-day culture (A) Representative fluorescent images of HULEC-5a and IMR-90 cells stained with live (green)/dead (red) assay. Scale bar = 200 μm. (B) Representative fluorescent images of HULEC-5a cells immunostained for VE-cadherin (red) and counterstained with DAPI (blue). Scale bar = 25 μm. (C) Apparent permeability of the ASTEROIDS membrane seeded with (orange) and without (black; control) HULEC-5a to FITC-5kDa dextran. *n* = 5 chips for each condition. (D) Expression level of shear stress-responsive candidate genes in HULEC-5a cells quantified by qRT-PCR 4 days after culture in the ASTEROIDS with flow and normalized to culture in Transwell with no flow. *n* = 3. (E) Representative bright field images of A549 spheroids taken directly from the ASTEROIDS at days 0, 3, and 7. Scale bar = 250 μm. Graph shows the surface areas of A549 spheroids normalized to day 0. *n* > 10 spheroids from 3 biological replicate chips. (F) Representative fluorescent images of A549 spheroids stained with live (green)/dead (red) assay. Scale bar = 250 μm. (G and H) Representative fluorescent images of A549 spheroids immunostained with Ki-67 (red) (G) and HIF-1α (red) (H) and counterstained with DAPI (blue). Scale bar = 250 μm. Data are represented as mean ± s.e.m. ∗*p* < 0.05, ∗∗*p* < 0.01, ∗∗∗∗*p* < 0.0001 by unpaired, two-tailed Student’s t-tests with a significance threshold of α < 0.05.

To assess if the ASTEROIDS could maintain a good cell viability in each compartment, we first co-cultured lung cancer spheroids (A549) in the central piece, with lung endothelial (HULEC-5a) and fibroblasts (IMR-90) cells seeded on the membrane of their respective chamber for 7 days. Data showed that both HULEC-5a and IMR-90 cells were alive after 7 days (Figure 2A). Interestingly, HULEC-5a cells also formed an intact monolayer linked by continuous junctional complexes containing the vascular endothelial cadherin (VE-cadherin) protein (Figure 2B and S2). The permeability of the membrane to FITC-dextran significantly decreased in the presence of HULEC-5a cells compared to a cell-free collagen-coated membrane, suggesting that the HULEC-5a monolayer effectively formed an endothelial barrier capable of decreasing diffusion of small molecules (Figure 2C). With perfused side chambers, the ASTEROIDS can also recapitulate blood flow and physiological mechanical forces applied to the endothelial cells. These hemodynamic forces are known to alter endothelial cell gene expression and to trigger a flow-mediated mechanotransduction response through the involvement of several molecular sensors. In particular, laminar shear stress has been shown to inhibit YAP/TAZ activity and pro-inflammatory response^16^^,^^17^^,^^18^ through integrin activation,^19^ to increase VEGF expression^20^ and to promote collagen production to help remodel the endothelial basement membrane.^21^ When compared to a static transwell culture, the expression levels of YAP/TAZ pathway genes (TAZ, CTGF, ANKRD1, and CYR61) and pro-inflammatory genes (CSF2, CCL2, and CXCL2) in HULEC-5a cells cultured in ASTEROIDS showed a decreased trend (Figure 2D). Conversely, VEGFR1/2, ITGA1/ITGB1, and COL1/4/6A1 expression increased in HULEC-5a cells cultured in ASTEROIDS, suggesting that HULEC-5a cells can sense the flow-induced mechanical forces. The A549 spheroids also showed the ability to grow with a projected area that increased > 2-fold over 7 days (Figure 2E). Live/dead staining showed a compartmentalized structure of the spheroids, with the presence of a necrotic core and live cells at the periphery, suggesting the presence of gas and nutrient gradients (Figure 2F). This observation was confirmed by the staining of the proliferation marker protein Ki-67 and hypoxia-inducible factor 1-alpha (HIF-1α), as evidenced by the existence of a proliferating edge (Figure 2G) and a central hypoxic area (Figure 2H), demonstrating that the culture of spheroids in the ASTEROIDS maintained their zonal architecture.

### Cell communication within the ASTEROIDS to recapitulate *in vivo* hallmarks

After demonstrating that the ASTEROIDS can maintain cell viability and key TME hallmarks, we aimed to assess whether the different compartments could communicate, especially between the central wells (cancer cells) and the chambers (stroma). Recent *in vivo* studies have investigated the transcriptomic profile of tumor endothelial cells, highlighting the heterogeneity of their phenotypes within the human TME.^22^^,^^23^ Interestingly, a study highlighted how human lung cancer cells alter endothelial cell behavior, enhancing angiogenesis and immune regulation, which supports tumor growth and metastasis within the complex TME.^24^ A key observation highlighted the differences between non-malignant and tumor-derived lung endothelial cells, revealing that Myc targets were the most enriched signature in tumor endothelial cells due to elevated transcription rates, while the most significantly downregulated pathway was associated with inflammatory responses.

**Figure 3:**
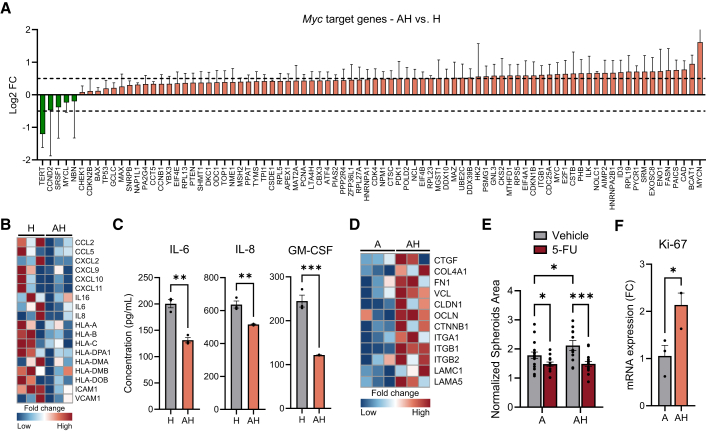
ASTEROIDS allows biochemical communication and reproduces *in vivo* cellular interactions (A) Log-transformed fold changes of Myc target genes expressed in HULEC-5a cells cultured in the ASTEROIDS in the presence of A549 spheroids (AH) vs. in the absence of A549 spheroids (H). *n* = 3. (B) Heatmap comparing the expression level of candidate immune genes expressed in HULEC-5a cells cultured in the ASTEROIDS in the absence (H) or presence of A549 spheroids (AH). *n* = 3. (C) Expression level of three cytokines in ASTEROIDS supernatant cultured for 3 days with HULEC-5a cells alone (H) or in co-culture with A549 spheroids (AH). *n* = 3. ∗∗*p* < 0.01, ∗∗∗*p* < 0.001 by unpaired, two-tailed Student’s t-tests with a significance threshold of α < 0.05. (D) Heatmap compares the expression level of genes involved in cell-cell and cell-ECM interactions expressed in A549 cells cultured in the ASTEROIDS in the absence (A) or presence of HULEC-5a cells (AH). *n* = 3. (E) Surface area of A549 spheroids normalized to day 0 after 4 days of culture in ASTEROIDS in the absence (A) or presence (AH) of HULEC-5a and after the injection of 5-fluorouracil (5-FU) or DMSO (Vehicle). *n* > 10 spheroids from 3 biological replicate chips. ∗*p* < 0.05, ∗∗∗*p* < 0.001 by two-way ANOVA with Fisher’s LSD test. (F) Ki-67 mRNA expression fold change in A549 spheroids when cultured in the absence (A) or presence (AH) of HULEC-5a in ASTEROIDS. *n* = 3. ∗*p* < 0.05 by unpaired, two-tailed Student’s t-tests with a significance threshold of α < 0.05. Data are represented as mean ± s.e.m.

To evaluate the cellular communication within the ASTEROIDS, we thus compared the expression levels of transcripts from Myc targets and inflammatory pathways in HULEC-5a cells when cultured in the absence or presence of A549 spheroids. Data showed that 79 out of 84 Myc target genes were overexpressed in HULEC-5a cells in the presence of A549 spheroids (Figure 3A). Conversely, expression levels of cytokines and chemokines transcripts, as well as genes involved in major histocompatibility complex I and II antigen presentation, were decreased in HULEC-5a cells co-cultured with A549 spheroids (Figure 3B). This observation was verified by the significantly lower levels of pro-inflammatory cytokines (IL-6, IL-8, and GM-CSF) in the supernatant of ASTEROIDS with HULEC-5a and A549 cells compared to ASTEROIDS with HULEC-5a alone (Figure 3C). Altogether, these data demonstrated that the presence of A549 spheroids altered the transcriptomics profile of lung endothelial cells that acquired a tumor endothelial cell-like phenotype, as observed *in vivo*.^25^ Moreover, this suggests that intercellular communication between the spheroids in the well and the cells seeded on the membrane effectively occurred within the ASTEROIDS.

To confirm this interaction, we then assessed if HULEC-5a cells could also modify the A549 spheroids' transcriptomic profile. Results showed the presence of HULEC-5a increased the expression of A549 transcripts involved in the extracellular matrix (ECM) organization and cell-cell and cell-ECM junctions (Figure 3D), highlighting the reciprocal interaction between the endothelial and cancer cells. Finally, to assess the capacity of small molecules to be perfused and to diffuse from the side chambers to the wells, we challenged the A549 spheroids by injecting the anticancer drug 5-fluorouracil (5-FU) in the vascular compartment, in the presence or absence of HULEC-5a. Data showed that the A549 spheroid surface area was significantly decreased in the presence of 5-FU compared to the control, suggesting that the 5-FU was able to pass the endothelial barrier and inhibit the growth of A549 spheroids (Figure 3E). The antiproliferative effect of 5-FU was confirmed by the decrease of Ki-67 expression in A549 spheroids exposed to the drug (Figure S3A). Previous studies have shown that 5-FU exposure also affects endothelial cells by inhibiting proliferation, affecting the cell cycle, and inducing senescence.^26^^,^^27^ Our data showed that the ASTEROIDS recapitulated this effect with a decrease of Ki-67 protein levels and VE-cadherin and claudin-5 mRNA expression as well as an increase in the expression of the cell cycle inhibitor and senescence biomarker CDKN1A in HULEC-5a cells exposed to 5-FU (Figures S3B–S3D).

Additionally, it is important to note that in the absence of 5-FU, A549 spheroids exhibited significantly larger areas when co-cultured with HULEC-5a cells compared to monoculture conditions, indicating that HULEC-5a cells may promote A549 spheroid growth (Figure 3E). This effect was confirmed by the increased expression of Ki-67 at both protein (Figure S3A) and mRNA (Figure 3F) levels in A549 spheroids cocultured in the presence of HULEC-5a cells, underlining the role of endothelial cells in tumor growth as previously described in *in vivo* studies.^24^ Together, these data confirmed the diffusion of soluble compounds within the ASTEROIDS and highlighted the communication between the cells on the membrane and the spheroids in the central wells.

### Integration and effect of the immune component within the ASTEROIDS

**Figure 4:**
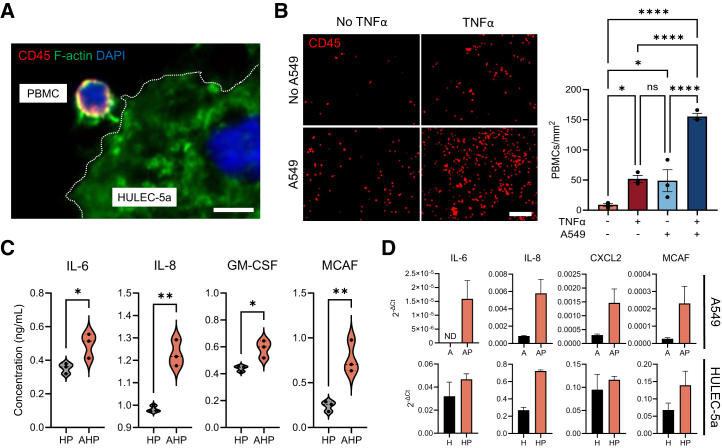
Immune cells flow in ASTEROIDS, interact and communicate with cancer and stromal cells (A) Fluorescent image of a CD45-positive cell (PBMC) interacting with a HULEC-5a cell seeded on ASTEROIDS membrane. Dotted line delineates the HULEC-5a membrane. Scale bar = 10 μm. (B) Representative fluorescent images of HULEC-5a membrane after 24 h of TNFα activation, in the absence or presence of A549 spheroids. Membranes have been stained with anti-CD45 to detect PBMC recruitment. Scale bar = 200 μm. The graph shows the number of PBMCs recruited per membrane area before and after TNFα induction, in the absence or presence of A549 spheroids. *n* = 3. ∗*p* < 0.05, ∗∗∗∗*p* < 0.0001, ns = non-significant by Fisher’s one-way ANOVA. (C) Comparison of cytokine levels in ASTEROIDS supernatant cultured for 3 days with HULEC-5a-PBMCs (HP) vs. A549-HULEC-5a-PBMCs (AHP). *n* = 3. ∗*p* < 0.05, ∗∗*p* < 0.01 by unpaired, two-tailed Student’s t-tests with a significance threshold of α < 0.05. (D) Gene expression level of cytokines in A549 (A) and HULEC-5a (H) cells cultured in ASTEROIDS in the absence or presence of PBMCs (AP/HP). ND = not detected. *n* = 3. Data are represented as mean ± s.e.m.

An important cellular component in the TME is the immune system, known to release cytokines, alter metabolism, remodel tissue, and engage in cell-to-cell signaling, thus shaping tumor growth, metastasis, and treatment response.^28^^,^^29^ Therefore, we aimed to assess whether the ASTEROIDS could also allow the perfusion of peripheral blood mononuclear cells (PBMCs) in the endothelial chamber and promote their interaction with the cancer and stromal cells. First, ASTEROIDS was primed with TNFα to activate the HULEC-5a cells, and after injection, PBMCs showed their ability to attach to HULEC-5a cells (Figure 4A) with a significant increase in attachment to activated cells (52 cells/mm^2^) compared to inactivated HULEC-5a cells (9 cells/mm^2^) (Figure 4B). Interestingly, the recruitment of PBMCs to endothelial cells was also increased in the presence of A549 spheroids (49 cells/mm^2^), with a significant synergistic effect under TNFα stimulation (155 cells/mm^2^), highlighting the existence of a PBMCs-A549 communication. To explain the increase in PBMC recruitment in the presence of A549 spheroids, cytokines in the ASTEROIDS supernatant were then quantified. Results showed that the levels of IL-6, IL-8, GM-CSF, and MCAF increased in ASTEROIDS cultured with HULEC-5a-PBMCs-A549 compared to ASTEROIDS cultured with HULEC-5a-PBMCs only (Figure 4C). Overall, the levels of IL-6, IL-8, CXCL2, and MCAF transcripts were increased in A549 cells and, to a lesser extent, in HULEC-5a cells when the cells were cultured in the presence of PBMCs (Figure 4D). While we cannot exclude the possibility that trace amounts of PBMC RNA contributed to these measurements, our protocol for sample recovery and previous observations suggest that such a contribution was minimal. Thus, the observed increases are most likely attributable to the interactions between A549 or HULEC-5a cells and PBMCs. Altogether, these data showed that the ASTEROIDS can integrate the immune component and that the cancer and stromal cells sensed the presence of immune cells.

### The ASTEROIDS as a radiation model for the tumor microenvironment

**Figure 5:**
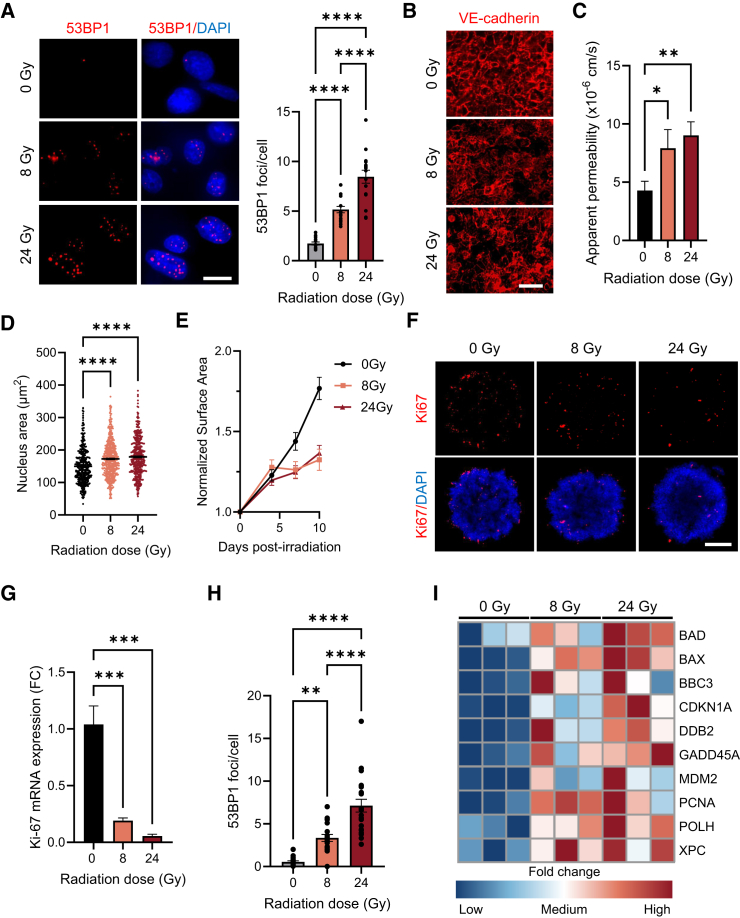
Cells within the ASTEROIDS display radiation-induced damages after SBRT-like radiation exposure (A) Representative fluorescent images of HULEC-5a cells 24 h after 0, 8, and 24 Gy-irradiation and immunostained for 53BP1. DAPI counterstaining is shown in blue. Scale bar = 20 μm. The graph shows the quantification of 53BP1 foci per cell for each condition. *n* = 15 frames with at least 100 nuclei each from 3 independent devices. (B) Representative fluorescent images of HULEC-5a cells irradiated at 0, 8, and 24 Gy and immunostained for VE-cadherin. Scale bars = 50 μm. (C) Apparent permeability of the ASTEROIDS membrane seeded with HULEC-5a to FITC-15kDa dextran 24 h post-irradiation compared to control (0 Gy). *n* = 3 chips for each condition. (D) Quantification of the nucleus area of HULEC-5a cells 24 h after 0, 8, and 24 Gy-irradiation. *n* > 400 nuclei from 4 independent devices. (E) A549 spheroids projected area measured over 10 days after 0, 8, and 24 Gy irradiation. *n* = 14 spheroids from 3 independent devices. (F) Representative fluorescent images of A549 spheroids irradiated at 0, 8, and 24 Gy and immunostained for Ki-67. DAPI counterstaining is shown in blue. Scale bar = 250 μm. (G) Ki-67 mRNA level in A549 spheroids quantified by qRT-PCR. *n* = 3. (H) Quantification of 53BP1 foci per cell in A549 spheroids 24 h after 0, 8, and 24 Gy-irradiation. *n* = 17 frames with at least 10 nuclei each from 4 independent devices. (See also Figure S4). (I) Heatmap shows the expression of radiation-responsive genes from p53 pathways in A549 spheroids 24 h after 0, 8, and 24 Gy-irradiation. *n* = 3. Data are represented as mean ± s.e.m. ∗*p* < 0.05, ∗∗*p* < 0.01, ∗∗∗*p* < 0.001, ∗∗∗∗*p* < 0.0001 by one-way ANOVA with Tukey’s multiple comparison test.

Finally, to determine whether the cells responded appropriately to radiation exposure, the ASTEROIDS, containing HULEC-5a cells, A549 spheroids, and PBMCs, were exposed to a single fraction of different stereotactic body radiation therapy (SBRT) doses (8 and 24 Gy) using a high energy 6MV photon beam. SBRT is an established treatment of choice for patients with early-stage, medically inoperable non-small cell lung cancer, offering high local control rates comparable to surgery.^30^^,^^31^ Fractional doses typically range from 7 to 12 Gy, with some protocols delivering up to 34 Gy per fraction in select cases.^32^ OoCs are particularly valuable for the investigation of new radiotherapeutic strategies as they offer a human-relevant and dynamic *in vitro* model, addressing the challenges of replicating the multifactorial human radiation response and the ethical constraints of exposing patients to new protocols. First, nuclear 53BP1 foci were quantified to assess DNA double-strand breaks (DSBs) on HULEC-5a.^33^^,^^34^ Data showed a significant dose-dependent rise in 53BP1 foci after 8 and 24 Gy (2-fold and 4-fold increases, respectively) compared to the non-irradiated control (Figure 5A). The function of the HULEC-5a barrier was then evaluated, and VE-cadherin staining showed a disruption of the cell-cell junctions after irradiation (Figure 5B), leading to an increase in barrier permeability (Figure 5C). Moreover, a significant increase in the nucleus area in irradiated HULEC-5a cells compared to non-irradiated cells was also observed, highlighting the radiation-induced nuclear morphological changes in endothelial cells (Figure 5D). To assess the radiation response of the cancer cells, the surface area of A549 spheroids was measured for 10 days post-irradiation. Data showed that the growth of irradiated A549 spheroids was highly inhibited compared to the control spheroids, whose surface area increased linearly (Figure 5E). This growth delay was confirmed by the reduction of Ki-67 expression in irradiated spheroids at both the protein and mRNA levels (Figures 5F and 5G), validating the decrease in proliferation activity of A549 cells post-irradiation. Like the HULEC-5a cells, the A549 spheroids displayed a dose-dependent increase in 53BP1 foci after irradiation (Figure 5H and S4), suggesting the initial formation of DSBs and presence of residual damages at 24 h. To confirm the cellular response to these residual radiation-induced damage, the expression of well-known radiation-responsive genes involved in the p53 pathway^35^^,^^36^ was assessed by qPCR. Results showed an upregulation of BAD, BAX, BBC3, CDKN1A, DDB2, GADD45A, MDM2, PCNA, POLH, and XPC transcripts, demonstrating that A549 spheroids responded to radiation by activating the p53-dependent DNA damage response (Figure 5I).

### Radiation-induced metabolomic changes in the ASTEROIDS

**Figure 6:**
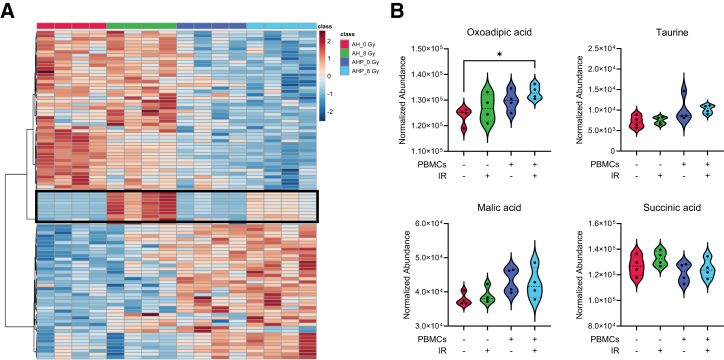
The presence of PBMCs alters the metabolomic profiles of the ASTEROIDS-simulated TME after radiation exposure Metabolomic analysis of culture media from the ASTEROIDS co-cultured with A549 (A) and HULEC-5a (H) cells, with or without PBMCs (P), and exposed to 0 or 8 Gy of X-rays. (A) A heatmap with the top 100 statistically significant ions highlights the importance of the immune system in modulating the availability of small molecules. Exposure to ionizing radiation also leads to changes in levels of such small molecules, most evident in the middle of the heatmap (black frame). *n* = 4. (B) Four validated metabolites with previously known association with radiation exposure were plotted and showed trends of increase following exposure to 8 Gy. *n* = 4. ∗*p* < 0.05 by one-way ANOVA with Tukey’s multiple comparison test.

Ionizing radiation induces metabolomic changes in the TME, including alterations in DNA repair mechanisms, fatty acid metabolism, redox balance, and overall metabolic reprogramming, which ultimately affect the stromal environment and modulate the tumor response.^37^ Immune cells play an important part in this response by altering cytokine production, adjusting to hypoxia, producing immunosuppressive metabolites, and remodeling the ECM, collectively leading to metabolic adaptation and shaping tumor progression.^38^ In addition, excreted metabolites can act as signaling molecules themselves and modulate cell survival and differentiation, and immune activation, among others.^39^ Therefore, we then aimed to investigate the radiation-induced metabolomic changes in the supernatant of ASTEROIDS and compare the response in the absence or presence of PBMCs to assess whether the platform could reproduce the metabolomics hallmarks of the irradiated TME. Data analysis through MetaboAnalyst 6.0 showed distinct changes in spectral features, with significant contributions from the PBMCs (Figure 6A). Exposure to 8 Gy of high energy X-rays also led to different clusters of altered levels in both groups (with and without PBMCs). Nine metabolites were validated with tandem mass spectrometry, and these metabolites were selected based on (a) biological significance, and (b) previously identified as metabolites whose levels may be altered by radiation exposure (Table S1). Only oxoadipic acid had a *p* < 0.05; however, trends were observed in the rest of the metabolites after radiation (Figure 6B). In addition, a distinct cluster of 9 spectral features was clearly increased in the irradiated group, with putative identifications showing potential involvement in pro-inflammatory processes or oxidized fatty acids that could result from lipid peroxidation and radiation-induced membrane damage (Table S2). Interestingly, these changes were less pronounced in the presence of PBMCs (Figure 6A, frame), suggesting a possible mitigating effect of the immune component on the TME early radiation response. The quantification of pro-inflammatory cytokines (IL-6, IL-8, GM-CSF, and MCAF) showed that although their levels increased in the presence of PBMCs pre-irradiation, their levels decreased post-irradiation in the presence of PBMCs only (Figure S5A). In addition, a comparative analysis of the radiation-induced nuclear hypertrophy showed that in the presence of PBMCs, the nuclear area of HULEC-5a cells after radiation was significantly lower compared to the nuclear area of HULEC-5a cultured without PBMCs (Figure S5B). Similarly, the number of radiation-induced 53BP1 foci was lower in both HULEC-5a and A549 cells when co-cultured with PBMCs compared to the culture of HULEC-5a+A549 in the absence of PBMCs (Figure S5C). Altogether, these data showed that the ASTEROIDS could recapitulate some key radiation-induced metabolomic changes and highlighted the role of the immune component in the regulation of these alterations.

## Discussion

The development of OoC must consider both biological and engineering factors to balance physiological relevance, measurement capabilities, and scalability to create effective tissue models.^5^ To this end, the engineering design of the ASTEROIDS confers several advantages. First, the ASTEROIDS device is made of polycarbonate, a thermoplastic polymeric material that provides superior manufacturability through injection molding, low evaporation, and resistance to small-molecule absorption, overcoming some of the main limitations of PDMS-like materials.^40^ Second, the device’s footprint, compatible with the 96-well plate format, facilitates integration with automated systems, enabling medium to high-throughput applications. Third, despite limited visualization of side channels, the optical clarity of the central wells permits direct live-cell microscopy imaging in a manner comparable to conventional 96-well plate formats. Fourth, the incorporation of a clamping system for non-destructive disassembly allows for easy removal of membranes and 3D tissues while preserving their structural integrity and cellular spatial organization. This feature also enhances the platform’s versatility, enabling accurate endpoint analyses such as high-resolution imaging and omics studies of cellular components, as well as offline measurements of the supernatant.

From a biological perspective, the ASTEROIDS proposes a unique configuration allowing the co-culture of 3D cellular structures with cellular monolayers seeded on membranes within perfused chambers. The spheroids are pre-formed using conventional culture and then transferred to the central wells, embedded in polymer gel, and, consequently, isolated from the fluid stress present in the side chambers. Therefore, this design differs from engineered microdevices that use microfluidics-guided spatial-temporal control and mechanical forces modeling to promote the generation and maintenance of spheroids.^41^^,^^42^ However, our results showed that the ASTEROIDS still maintain the compartmentalized structure of the spheroids, suggesting that it did not widely alter their phenotype and provided good conditions for the study of 3D cellular structures. The ASTEROIDS side chambers maintained spatial segregation of stroma cells seeded on the membrane from the spheroids, thus providing a tissue barrier and a hollow vasculature, enabling the recapitulation of long-term perfusion conditions. This is also different from some spheroids-on-chip that promote sprouting angiogenesis for a direct vascularization of the 3D cellular structures.^43^^,^^44^^,^^45^ The potential for angiogenesis in ASTEROIDS has not been empirically evaluated; although it could theoretically be feasible with specific design modifications, including increasing the diameter of membrane pores or reducing the spatial gap between the spheroids and the membrane interface. Similarly, while we observed adhesion of PBMCs to the endothelial monolayer under flow, we did not assess whether they could transmigrate across the membrane and access the central well containing the spheroids. We anticipate that such extravasation would be feasible in the ASTEROIDS platform, potentially through design modifications analogous to those proposed for promoting angiogenesis. Evaluation of this process should be a priority for future investigations, as PBMC trafficking within the TME represents a critical determinant of tumor progression and therapeutic response. Nevertheless, even without direct contact, we demonstrated the alteration of the transcriptomic profiles of spheroids and endothelial cells during co-culture compared to monoculture, as well as the effect of small compounds (i.e., 5-FU) injected in the vascular chamber. This observation demonstrates the presence of intercellular communication between the membrane and the wells, likely mediated by paracrine signaling and the diffusion of soluble factors.

With its unique combination of 3D cancer tissue interfacing with up to 3 different cell types, the ASTEROIDS reaches a certain degree of complexity that could potentially achieve high-fidelity replication of tumor heterogeneity and stromal interactions, enabling more accurate prediction of clinical responses and personalized therapeutic strategies.^46^ In this feasibility study, we demonstrated that the ASTEROIDS maintained long-term cell viability, allowed cellular communication, immune interaction, and response to drugs and radiation, suggesting that the model can reproduce key aspects of the tumor-level physiology. Future research should explore the potential of ASTEROIDS in modeling additional processes such as cancer invasion, metastasis, immunotherapy screening, and its predictive capacity for personalized medicine. The versatility of the platform could also be tested for the investigation of other systems and organs beyond the TME, particularly those requiring this level of complexity, including 3D structures, tissue interfaces, and immune components.

The complexity of the radiation response and its systemic effect on a broad range of tissues require advanced *in vitro* models to understand the mechanisms involved in the human TME during radiation treatment. Since intentionally exposing healthy humans to radiation is ethically not feasible, OoC technology has gained attention as a promising advanced model for human radiobiological research.^47^^,^^48^^,^^49^^,^^50^^,^^51^ In recent years, gut-,^52^ bone marrow-,^53^ lung-,^54^ vessel-,^55^ and even multi OoC^56^ have been exposed to different radiation regimens and qualities. These studies demonstrated their ability to reproduce key radiotoxicity hallmarks and utility to test countermeasure drugs. Similarly, our study showed that the irradiation of the ASTEROIDS resulted in a nuclear hypertrophy, disruption of adherens junctions, and permeabilization of the endothelial barrier, mimicking the radiation-induced vascular leak observed *in vivo.*^57^^,^^58^ A dose-dependent increase in residual DNA damage and inhibition of spheroid growth have also been observed after SBRT, suggesting that the ASTEROIDS recapitulates tumor radiobiology and clinical data of SBRT.^59^ Metabolic changes in the TME are increasingly recognized as influential in cancer development and response to treatment, providing insights into key pathways and opportunities for therapeutic advancements.^60^^,^^61^ Ionizing radiation is also known to affect the metabolic profile of both normal tissue and TME,^62^^,^^63^ which can lead to immunomodulation for treatment enhancement. However, this metabolic response to radiation remains poorly understood, necessitating further investigation to elucidate its mechanisms. In this study, we profiled circulating media from irradiated ASTEROIDS and showed the platform’s capability to explore radiation-induced metabolic alterations. Our analysis focused only on the analysis of the supernatant, representative of the changes observed in biological fluids and of tissue responses to radiation; however, future directions should incorporate both supernatant and cellular analysis for a more comprehensive analysis. Nevertheless, this profiling underscored the critical role of PBMCs in the radiation-induced regulation of the TME metabolism, potentially conferring radioprotective effects, and more importantly, it recapitulated similar responses with other endpoints, as shown in this study. This underscores the importance of incorporating immune components to enhance the physiological relevance and predictive value of experimental radiobiological models, as the absence of such protective effects may lead to exaggerated *in vitro* responses that fail to accurately reflect the more attenuated outcomes observed *in vivo*. Moreover, the impact of radiation therapy on the immune system within the TME is complex, eliciting either stimulatory or suppressive responses and varying based on the type of cancer. The factors governing these divergent immune outcomes are not yet clear, including, for example, the influence of radiation dose (low, high, cumulative) and/or fractionation schemes. Our preliminary data showed that the integration of the immune component into the multicellular environment of the ASTEROIDS could provide a new tool for addressing these unresolved questions.

Altogether, these experimental data provide compelling evidence for the efficacy of ASTEROIDS in recapitulating viable cellular interactions between endothelial, immune cells, and 3D cellular structures, analogous to *in vivo* observations. These interactions then allowed for the reproduction of the phenotypic response to radiation exposure. This study establishes a foundation for future investigations to elucidate the underlying biological mechanisms involved in multiple radiation scenarios, and, more importantly, represents a direct human-to-3D tissue model, effectively leading to new avenues of personalized medicine.

### Limitations of the study

As stated previously, the present study served as a proof of concept that aimed to demonstrate the ability of the ASTEROIDS device to maintain viable 3D multicellular cultures in a dynamic environment. The device was conceived as a user-oriented system that integrates key features designed to facilitate adoption by end-users. Biologically, the data showed that the ASTEROIDS reproduced key hallmarks of the TME, such as zonal tumor architecture, endothelial barrier formation, and cross-communication between immune, endothelial, and tumor compartments. Notably, the system also reproduced the TME’s response to external stimuli such as radiation therapy. Although these preliminary data support the concept, several limitations should be acknowledged. From an engineering standpoint, the ASTEROIDS device is fabricated entirely from polycarbonate, which provides structural stability but reduces deformability^5^ and limits the ability to incorporate dynamic motions, such as the stretching that could be used to mimic lung breathing.^64^ Although the material’s optical transparency allows direct visualization of 3D constructs, live imaging of cells located in the lateral chambers remains inaccessible. Moreover, the 2 mm bottom thickness of ASTEROIDS is substantially greater than that of specialized imaging plates (∼0.2 mm), potentially reducing compatibility with high-resolution objectives and necessitating the use of long-working-distance lenses, which generally offer lower numerical apertures and thus lower maximal image resolution. The integration of lenses and optical systems could be explored in the future to enable *in situ* imaging at high resolution. From a biological perspective, additional considerations emerge. First, although the ASTEROIDS supports 3D tissue growth, the spheroids must be preassembled externally, are not exposed to fluid stress, and require hydrogel embedding to preserve their structural integrity. Second, cell-cell communication occurs solely at a distance through secreted messengers and does not involve direct cell-cell contact, yet is essential in many fundamental biological processes, including tumorigenesis.^65^^,^^66^ Finally, this proof-of-concept study was conducted using established cell lines, and the use of pluripotent stem cell-derived or patient-derived cells would be required to fully assess the potential of the ASTEROIDS to faithfully recapitulate TME and, more broadly, the biological mechanisms underlying tissue homeostasis and disease pathology.

## Resource availability

### Lead contact

Requests for further information and resources should be directed to and will be fulfilled by the lead contact, Jerome Lacombe (jlacombe@arizona.edu).

### Materials availability

The devices generated in this study will be made available on request, but we may require a payment and/or a completed materials transfer agreement.

### Data and code availability

• All data reported in this article will be shared by the lead contact upon request. The metabolomics data supporting this publication are available at ImmPort (https://www.immport.org) under study SDY3333.
• This article does not report original code.
• Any additional information required to reanalyze the data reported in this article is available from the lead contact upon request.

## Acknowledgments

This study was supported by Mitsubishi Gas Chemical Company, the St. Joseph's Foundation, and the Center for Applied Nanobioscience and Medicine. The authors would like to thank the 10.13039/100019915Helios Education Foundation and the Valley Research Partnership Program (P1B-6027) for their support of undergraduate and graduate programs. F.Z. also acknowledges support from Honor Health Research Institute Rare Cancer Initiative, supported by Desert Mountain CARE. The metabolomics part of the project was also supported by an award no. P30 CA051008 (P.I. Louis Weiner) from the 10.13039/100000054NCI. F.Z., E.L., A.F Jr., and J.L. were partially supported by award no 1U01AI148307-01 (MPI Laiakis and Zenhausern) from 10.13039/100000060NIAID. The content is solely the responsibility of the authors and does not necessarily represent the official views of the 10.13039/100000002NIH.

The authors would also like to thank all the members of the Center for Applied Nanobioscience and Medicine for their support and contributions. We extend our gratitude to Dr. Kurt Gustin of the Biomedical Imaging Core at the UA College of Medicine − Phoenix for providing epifluorescent imaging services and to Dr. Timothy Marlowe, director of the Molecular Discovery Core at the UA College of Medicine – Phoenix, for providing fluorescence biomolecular imaging services. We also acknowledge Drs. Alan Gillman, Eric Bridenbaugh, and William JB Vincent for their expertise in acquiring confocal microscopy images of spheroids within the ASTEROIDS. Finally, we thank Messrs. Kirino Tomoaki and Ohno Daisuke from Mitsubishi Gas Chemical Company, Inc., for their help with the development and manufacturing of the ASTEROIDS platform.

## Author contributions

Conceptualization, J.L. and F.Z.; data curation, J.L., S.E.D., J.H., and E.C.L.; formal analysis, J.L., S.E.D., and E.C.L.; funding acquisition, J.L., S.F., A.D., and F.Z.; investigation, J.L, S.E.D., M.L., M.S., N.M.F., B.R., K.C., M.W.B., J.H., E.C.L., S.J., and S.S.; methodology, J.L., S.E.D., J.H., S.J., and S.S.; project administration, J.L. and F.Z.; resources, E.C.L., A.J.F., S.F., A.D., and F.Z.; supervision, J.L. and F.Z; visualization, J.L., M.W.B., J.H., and E.C.L.; writing – original draft, J.L., S.E.D., E.C.L, S.J., and S.S.; writing – review and editing, J.L., S.E.D., E.C.L, A.J.F., S.J., S.S, S.F., A.D., and F.Z.

## Declaration of interests

J.L., S.E.D., M.W.B., J.H., and F.Z. are inventors on US Patent Application No. 17/622,147 and PCT Application No. PCT/US24/46777. All other authors declare no competing interests.

## STAR★Methods

### Key resources table

**Table:** 

REAGENT or RESOURCE	SOURCE	IDENTIFIER
**Antibodies**		

Anti-53BP1	Abcam	Cat# ab21083; RRID:AB_722496
Anti-CD45	Thermo Fisher Scientific	Cat# 14-0459-82; RRID:AB_467274
Anti-HIF-1α (28b)	Santa Cruz Biotechnology	Cat# sc-13515; RRID:AB_627723
Anti-Ki67	Abcam	Cat# ab15580; RRID:AB_443209
Anti-VE-cadherin (F-8)	Santa Cruz Biotechnology	Cat# sc-9989; RRID:AB_2077957
Cy3-AffiniPure Goat Anti-Mouse IgG	Jackson ImmunoResearch Labs	Cat# 115-165-062; RRID:AB_2338685
Alexa Fluor 647-AffiniPure Goat Anti-Rabbit IgG	Jackson ImmunoResearch Labs	Cat# 111-605-045; RRID:AB_2338075

**Biological samples**		

Peripheral blood mononuclear cells	This paper	N/A

**Chemicals, peptides, and recombinant proteins**		

VitroGel® Organoid Recovery Solution	The Well Bioscience	Cat# MS04-100
Collagen I, rat tail	Thermo Fisher Scientific	Cat# A1048301
Matrigel® Matrix for Organoid Culture, Phenol Red-free, LDEV-free	Corning	Cat# 356255
DAPI	Thermo Fisher Scientific	Cat# D1306
5-Fluorouracil	Sigma-Aldrich	Cat# F6627
Dimethyl Sulfoxide	Fisher Scientific	Cat# BP231-100
MCDB 131 Medium, no glutamine	Thermo Fisher Scientific	Cat# 10372019
Fetal Bovine Serum	Thermo Fisher Scientific	Cat# 6140-071
Ham’s F-12K (Kaighn’s) Medium	Thermo Fisher Scientific	Cat# 21127-022
Penicillin-Streptomycin (10,000 U/mL)	Thermo Fisher Scientific	Cat# 15140-122
L-Glutamine	Thermo Fisher Scientific	Cat# 25030081
HyClone™ Dulbecco’s Phosphate Buffered Saline	Cytiva	Cat# SH30028.FS
Ficoll-Paque™ PLUS density gradient media	Cytiva	Cat# 17144002
Agar	Sigma-Aldrich	Cat# A9915
Bovine Serum Albumin	Sigma-Aldrich	Cat# A9647
Fluorescein isothiocyanate–dextran	Sigma-Aldrich	Cat# FD4
Acetonitrile Optima^TM^ LC/MS	Fisher Scientific	Cat# A955-4
Water Optima^TM^ LC/MS	Fisher Scientific	Cat# W6-4
Formic acid	Fisher Scientific	Cat# A11710X1-AMP
Methanol^TM^ LC/MS	Fisher Scientific	Cat# A456-4

**Critical commercial assays**		

Live/Dead Double Staining Kit	Sigma-Aldrich	Cat# QIA76
RNeasy Mini Kit	Qiagen	Cat# 74104
RT^2^ First Strand Kit	Qiagen	Cat# 330401
RT^2^ Profiler PCR Array Human MYC Targets	Qiagen	GeneGlobe ID: PAHS-177Z
Custom RT^2^ PCR Array	Qiagen	Cat# 330171
QuantiTect Reverse Transcription Kit	Qiagen	Cat# 205311
QuantiNova SYBR Green PCR Kit	Qiagen	Cat# 208054
Multiplex Human Cytokine ELISA Kit	MyBioSource	Cat# MBS590064

**Deposited data**		

Metabolomics data	https://www.immport.org	SDY3333

**Experimental models: Cell lines**		

A-549	ATCC	Cat# CCL-185; RRID:CVCL_0023
IMR-90	ATCC	Cat# CCL-186; RRID:CVCL_0347
HULEC-5a	ATCC	Cat# CRL-3244; RRID:CVCL_0A11

**Oligonucleotides**		

Primers for RT-qPCR, see Table S3	Eurofins Genomics	N/A

**Software and algorithms**		

SolidWorks Premium 2025	https://www.solidworks.com/	Version SP1.2
COMSOL Multiphysics	https://www.comsol.com/	Version 6.2
Aria/Eclipse	https://www.varian.com/	Version 15.6
Mobius3D	https://www.varian.com/	Version 4.0.2
ZEISS ZEN Microscopy Software	https://www.zeiss.com/microscopy/us/products/software/zeiss-zen.html#	Version 4.5
MxPro QPCR Software	https://www.agilent.com/	MxPro-Mx3005P v4.0
MetaboAnalyst	https://www.metaboanalyst.ca/	Version 6.0
Progenesis QI (NonLinear Dynamics)	http://www.nonlinear.com/progenesis/qi/	–
Microsoft Excel	https://www.microsoft.com/en-us/microsoft-365	Microsoft 365
ImageJ	https://imagej.net/ij/	Version 1.52a
GraphPad Prism	https://www.graphpad.com/	Version 10.4.1

**Other**		

Polycarbonate resin	Mitsubishi Engineering-Plastics Corp	Cat# lupilon^TM^ S-3000R
Silicone Rubber Compound for O-ring	Momentive Performance Materials	Cat# TSE221-5U
PET Sheet	AXEL Corporation	Cat# 2-3993-01
8.0 μm Hydrophilic Polycarbonate Track Etched Membrane Sheets	GVS North America, Inc.	Cat# 3033093
PharmedBPT pump tubing	Welco Co.	Cat# 8413.91
SILASTIC Laboratory Tubing	Cole Parmer	Cat# EW-96115-12
0.2 μm Cytiva Nanosep MF centrifugal device	Fisher Scientific	Cat# 50-197-9571
Phillips BigBore CT scanner	https://www.usa.philips.com/	–
Varian TrueBeam Linear Accelerator	https://www.varian.com/	version 3.0

### Experimental model and study participant details

#### Cell lines

Cell lines were obtained from and authenticated by the American Type Culture Collection (ATCC). The HULEC-5a cells (CRL-3244) were maintained in MCBD 131 [1X] [-] L-Glutamine media supplemented with 10% fetal bovine serum (FBS; Gibco), 10 mM L-Glutamine (Gibco), and 1% Penicillin-Streptomycin (P/S; 10 000 U/mL, Gibco). IMR-90 (CCL-186) and A549 (CCL-185) cells were maintained in F-12K [1X] media supplemented with 10% FBS and 1% P/S. All cell lines were maintained at 37°C in a 5% CO2 incubator between passages 4 through 10, and the culture media was changed every 48–72 h. Mycoplasma surveillance was performed monthly (MycoAlert, Lonza).

#### Human participants

Peripheral whole blood samples were collected by venipuncture on arms in heparin tubes from healthy donors (3 males, 27–39 years old, each used for one independent experiment) under approval of the University of Arizona Institutional Review Board (IRB-1708743060), after informed written consent was obtained from all participants. This data does not evaluate associations with sex or gender.

### Method details

#### Fabrication and assembly of the ASTEROIDS device

The central piece and perfusion chamber were fabricated from optically transparent, injection-molded polycarbonate (PC) using a precision-machined steel mold insert with defined geometric features at Mitsubishi Gas Chemical (Japan). Briefly, PC pellets (lupilon S-3000R) were dried at 120°C for 4 h prior to use, then melted and injected into the mold cavity at 260°C–285°C under pressures of 140–160 MPa. Mold temperature was maintained at 130°C to ensure complete replication of microstructures. Following cooling to below 80°C for 20–30 s, substrates were demolded and trimmed. Prior to assembly, each piece was autoclaved for 20 min at 121°C. Then, before mounting the perfusion chambers and the membranes on each side of the central piece, the central piece was first placed into a supporting holder to seal each side and fill each well with 65 μL of boiling 1.5% agarose diluted in PBS. A comb was then immediately placed on top for 15 min at room temperature to create a cavity in the agarose during polymerization, which would later hold the spheroids in place during culture and prevent their attachment to the plastic and subsequent disaggregation. After polymerization, the central piece was taken out from the holder and the comb removed, before two rectangular silicone (Momentive Performance Materials, TSE221-5U) O-rings were placed on each side of the central piece, followed by an 8-μm hydrophilic PC track-etched membrane (9–12 μm thickness) attached to a polyethylene terephthalate (PET) cutout (AXEL Corporation, 2-3993-1) covering the full side of the central piece. Two additional rectangular silicone O-rings were then positioned on each side of the perfusion chambers to ensure complete sealing and the chambers were clamped to the central piece with two PC butterfly keys (Figure 1A). After assembly, the devices were filled with Dulbecco’s Phosphate-Buffered Saline (DPBS), irradiated at 300 Gy using an X-ray cabinet (X-RAD 320, Precision X-ray Inc., North Branford, CT) and stored at 4°C for up to 2 weeks.

#### ASTEROIDS cell culture

Peripheral blood mononuclear cells (PBMCs) were immediately purified after whole blood collection by density gradient centrifugation (Ficoll-Paque PLUS, Cytiva) according to the manufacturer’s recommendations and resuspended in A549:HULEC-5a (1:1) medium. For the formation of spheroids, A549 cells were seeded at a density of 4000 cells/well in 96-well Ultra-low Attachment Plates (S-bio, MS-9096UZ) and transferred to the ASTEROIDS at day 4. Before inoculation of the cells in the ASTEROIDS, the pump tubing (PharmedBPT, Welco, 8413.91), the platinum-cured silicon tubing (Cole Parmer, EW-96115-12), connectors, and media glass bottles were cleaned with 100% bleach, flushed and boiled in deionized water. The components were then autoclaved for 20 min at 121°C. In the meantime, 500 μL of rat type I collagen (ThermoFisher, A1048301, diluted to a final concentration of 50 μg/mL in 0.02 M acetic acid) was dispensed into each perfusion chamber to generate a coating that facilitates cell adhesion. After 1 h incubation at 37°C, unbound collagen was washed out with 2 mL DPBS. Collagen-coated devices filled with DPBS can be stored up to 1 week at 4°C until use. For cell seeding, with the ASTEROIDS lying on the side, 500 μL of HULEC-5a cells at a concentration of 1.6.10^6^ cells/mL (∼3.10^3^ cells/mm2) were injected by pipetting in the vascular chamber and allowed to attach for 1 h at 37°C. The ASTEROIDS were then inverted, and the 500 μL of IMR-90 cells at a concentration of 1.8.10^6^ cells/mL were perfused by pipetting in the other chamber and allowed to attach for 1 h at 37°C. Finally, spheroids were seeded at a density of 1–6 spheroids/well, depending on the experiment, in the central piece before being covered with 18 μL of cold Matrigel (Corning, 356255) to provide ECM-like microenvironment. After 10 min incubation at room temperature to allow Matrigel polymerization, 100 μL of A549 complete medium was added into each well, while 500 μL of respective media were fed by pipetting into the perfusion chambers. The ASTEROIDS were then sealed on top by an adhesive PC lid to avoid leakage during perfusion and maintained under static conditions at 37°C overnight. On day 2, the ASTEROIDS were connected to the peristaltic pumps (Welco, WPM2-P3EA-WP) and media bottles, and the chambers were perfused at flow rate of 60 μL/min for the time of the experiment. For experiments longer than 4 days, medium in the media bottle was replaced at day 4 and every other day. Injection of PBMCs into the ASTEROIDS for all related experiments occurred on day 4. Briefly, after disconnecting the ASTEROIDS from the fluidic platform, 500 μL of PBMCs resuspended in A549:HULEC-5a (1:1) medium at a density of 1.10^6^ cells/mL were injected by pipetting directly in the vascular chamber. The ASTEROIDS was then connected back to the pump and PBMCs were perfused at flow rate of 60 μL/min for the rest of the experiment.

#### Computational fluid dynamic simulation

Fluid dynamic analysis was performed using COMSOL Multiphysics (COMSOL Inc., version 6.2). The ASTEROIDS side chamber geometry was imported from SolidWorks software (SolidWorks, 2025) and a physics-controlled mesh was created with an element size set to normal due to the limitations of both the computational capacity and processing time of the computer. The simulation was performed with single-phase creeping flow physics (suitable for microfluidics systems with small Reynolds number where inertia term can be neglected) to solve the incompressible Navier–Stokes equation and the continuity equation for the evaluation of the velocity field and the shear stress inside the ASTEROIDS side chamber. The steady-state condition was selected, with a no-slip boundary condition applied to the walls. A flow rate of 60 μL/min was used as inlet condition and a pressure = 0 atm (no backflow) was set at the outlet. Additionally, water at a temperature of 37°C was considered as a reference fluid to run the simulations as its Newtonian fluid properties are very similar to cell culture medium.

#### Immunofluorescence imaging

At the conclusion of all experiments, before disassembly, all medium and circulating cells were first flushed from the ASTEROIDS device. The ASTEROIDS device was then disassembled by unclipping the butterfly keys to remove the perfusion chambers and directly access the membranes and spheroids. The PET sheet was then carefully detached with tweezers, after which the polycarbonate (PC) membranes were excised using scissors and subsequently rinsed with DPBS. After membrane removal, 200 μL of DPBS was pipetted into the central wells to flush out the spheroids embedded in Matrigel. The Matrigel matrix was then dissolved using Vitrogel Organoid Recovery Solution according to the manufacturer’s specifications (The Well Bioscience, MS04-100). For the live/dead analyses, the cells were stained with the CalBiochem Live/Dead Double Staining Kit (Millipore) following manufacturer’s recommendations. For the immunostaining, the cells were fixed in 4% paraformaldehyde for 15 min and permeabilized with 0.1% Triton X-100/DPBS for 5 min at room temperature. After two washes in 0.05% Tween 20/DPBS, cells were blocked in 1% BSA/DPBS for 1 h at room temperature. Samples were then incubated for 1 h with antibodies diluted in 1% BSA/DPBS against 53BP1 (Abcam, ab21083, 1/400), HIF-1α (Santa Cruz, sc13515, 1/50), VE-cadherin (Santa Cruz, sc9989, 1/50), Ki-67 (Abcam, ab15580, 1/1000), or CD45 (Invitrogen, 14-0459-82, 1/200) followed by Cy3-conjugated anti-mouse IgG (Jackson ImmunoResearch, 115-165-062, 1/400) or Alexa Fluor 647-conjugated anti-rabbit IgG (Jackson ImmunoResearch, 111-605-045, 1/200) and counterstained with DAPI (Invitrogen, D1306, 300 nM). After mounting samples on microscope slides with the addition of antifade mounting medium, they were flattened with glass coverslip, and images were obtained using an FLUOVIEW FV4000 confocal laser scanning microscope or a Zeiss Axio Imager M2 epifluorescent microscope with a Zeiss AxioCam MRm camera using ZEN 4.5 software at the Biomedical Imaging Core Facility at the UA College of Medicine – Phoenix.

#### Permeability assay

To characterize the HULEC-5a cellular barrier function, a fluorescent dextran diffusion assay was performed. The ASTEROIDS were cultured with or without HULEC-5a cells only in one side chamber for 3 days. The well in the central piece and the opposite side chamber were incubated in culture media only. Devices were then prepped for the permeability assay by removing the side chamber perfused with only culture media, and a rubber gasket with clamp was applied to the respective center block side wall. The culture media of center block wells were aspirated and 150 μL of fresh culture media was then added to each well. Fluorescein isothiocyanate-conjugated 5 kDa dextran (Sigma-Aldrich) was diluted in culture media at a final concentration of 250 μg/mL. The diluted dextran solution was then perfused through the vascular chamber at 60 μL/min using a syringe pump. After 10 min perfusion, media from the center well was collected, and the fluorescence intensity measured using a CLARIOstar Plus microplate reader (BMG Labtech, Germany) at 483(Ex)/530(Em). The concentration of each well was determined by extrapolating unknown values from a standard curve. First, fluorescence of different concentrations of fluorescent dextran (250–0.122 μg/mL) was measured. A negative control made with medium only (no fluorescent dextran) was also included to measure fluorescence background. Data points were then plotted on a graph and a standard curve was generated using a linear regression of equation y = ax by subtracting the negative control to each data points and forcing the curve to pass through the coordinate point (x = 0; y = 0). The concentration x for each well was then calculated using the formula x = y/a derived from the linear regression of the standard curve where y is the measured fluorescence and a the slope of the standard curve. The diffusion of the fluorescent dextran from the vascular chamber to the well was then assessed by calculating the average apparent permeability (P_e_) of all wells using the equation:

$$P_{e}=\frac{Ct.V}{C_{0}.A.\Delta t}$$

where Ct is the concentration in the well, V the volume of the well, C_0_ the initial concentration, A the surface area between the vascular chamber and the well, and Δt the time of perfusion.

#### Transwell culture of HULEC-5a cells

Custom 12-well plate Transwell inserts with a bottom diameter of 12 mm were 3D printed according to manufacturer’s specifications. PC membranes with 8 μm pores (Whatman, 110614) were attached to the bottom of the insert using medical grade double sided adhesive (Adhesives Research, 90106NB) cut to the dimensions of the insert bottom lip. The insert’s membranes were then coated with collagen (diluted to a final concentration of 50 μg/mL in 0.02 M acetic acid) as described above for the ASTEROIDS membranes. After DPBS wash to remove unbound collagen, inserts’ membranes were seeded at a cell density of 3.10^3^ HULEC-5a cells/mm^2^, identical to the ASTEROIDS vascular chamber, in an A549:HULEC-5a (1:1) medium supplemented with Matrigel at a 12:1 ratio. RNA from HULEC-5a cells in both insert and ASTEROIDS was collected after 4 days of culture.

#### 5-Fluorouracil (5-FU) assay

ASTEROIDS were seeded with A549 and HULEC-5a cells, as described previously. Powdered 5-Fluorouracil (Sigma-Aldrich, F6627) was dissolved in DMSO (Fisher, BP231-100) to a final concentration of 300 mM and filter-sterilized. After 4 days of incubation, 5-FU was injected in the media bottle to a final concentration of 400 μM. The vehicle control consisted of cell culture media containing 0.06% DMSO, matching the concentration used in the drug-treated devices. Spheroid growth was monitored by taking pictures on day 3 post-injection using bright-field phase contrast microscopy.

#### Real-time quantitative reverse transcription PCR (qRT-PCR)

Spheroids recovered from the ASTEROIDS device following Matrigel removal, together with membranes detached from the PET sheet, were collected into 1.5 mL microcentrifuge tubes, and lysed in Buffer RLT Plus (Qiagen, 1053393) supplemented with 1% β-mercaptoethanol. RNA was then extracted using the RNeasy Mini Kit (Qiagen, 74104) according to the manufacturer’s recommendations. RNA quantification was performed using an Epoch microplate spectrophotometer (BioTek Instruments) and stored at −80°C until further use.

For the Myc target genes and inflammatory pathways genes, qRT-PCR was performed using RT_2_ Profiler PCR Array Human MYC Targets (Qiagen, #330231) and a custom RT_2_ Profiler PCR array (including CCL5, CXCL9, CXCL10, CXCL11, IL16, HLA-A, HLA-B, HLA-C, HLA-DPA1, HLA-DMA, HLA-DMB, HLA-DOB, GADD45A, MDM2, POLH, XPC), respectively. Three hundred nanograms of total RNA were first used to generate cDNA with the RT_2_ First Strand Kit (Qiagen) according to manufacturer’s instructions. Briefly, the reaction was first incubated at 42°C for 5 min to eliminate residual genomic DNA and placed 1 min on ice. After addition of the reverse transcription mix, reaction was placed at 42°C for 15 additional minutes and stopped by incubation at 95°C for 5 min before proceeding to PCR. The cDNA was then added to the RT_2_ Profiler PCR 96-well plate along with the RT Mastermix containing 2X RT_2_ SYBR Green. PCR reactions were carried out using a Stratagene Mx30005P (Agilent Technologies, Inc.). Cycling parameters were 10 min at 95°C for initial denaturation, followed by 40 cycles of denaturation at 95°C for 15 s and annealing and extension at 60°C for 1 min. Melting curves were automatically generated, ranging stepwise from 60°C to 95°C and data was collected by MxPro qPCR Software (Agilent).

For the other targeted genes, 100 ng of total RNA was first used to generate cDNA using the QuantiTect Reverse Transcriptase kit (Qiagen, 205311) according to the manufacturer’s instructions. Briefly, the reaction was incubated at 42°C for 2 min to eliminate residual genomic DNA and then placed immediately on ice. After addition of the reverse transcription mix, the reaction was placed at 42°C for 15 additional minutes and stopped by incubation at 95°C for 3 min before proceeding to PCR using the QuantiNova SYBR Green PCR kit (Qiagen, 208054). The primers were synthesized by Eurofins Genomics and their sequences are provided in Table S3. Cycling parameters were 2 min at 95°C for initial activation, followed by 40 cycles of denaturation at 95°C for 5 s and combined annealing and extension at 60°C for 10 s. Melting curves and data were collected as previously described. Values were normalized with glyceraldehyde-3-phosphate dehydrogenase (GAPDH) for A549 cells and hypoxanthine phosphoribosyltransferase 1 (HPRT1) for HULEC-5a cells, and analyzed according to the ΔΔCt method.^67^

#### Cytokine analysis

Effluents were collected 4 days after cell loading, immediately centrifuged at 3,900 g for 10 min to remove cellular debris and stored at −80°C until analysis. A volume of 100 μL from each sample was then analyzed for 6-panel of pro-inflammatory cytokines (IL-1α, IL-1β, IL-6, IL-8, GM-CSF, IFN-γ, MCAF, TNFα) using the Multiplex Human Cytokine ELISA Kit (MyBioSource, MBS590064) according to the manufacturer’s standard protocol. Colorimetric signal was measured at 450 nm using an Epoch microplate reader (BioTek Instruments).

#### Irradiation

The ASTEROIDS devices were irradiated using a TrueBeam C-arm linear accelerator (Varian, Palo Alto, CA) utilizing a 6 MV energy and 600 MU/min dose rate at the Dignity Health Cancer Institute at St. Joseph’s Hospital and Medical Center (Phoenix, AZ). Three assembled ASTEROIDS, filled with a medium with similar density characteristics as the seeded cell lines described above, were placed side-by-side on top of a 5 cm slab of solid water (Sun Nuclear, Melbourne, FL). Custom pieces of 0.5 cm Superflab bolus (Radiation Products Design, Inc., Albertville, MN) were placed on top and the sides of the ASTEROIDS to allow sufficient dose buildup at the level of the seeded wells. A treatment planning scan was obtained using a Brilliance Big Bore CT scanner (Philips, Amsterdam, Netherlands) using a technique, scan length, and field of view appropriate to the ASTEROIDS dimensions. A slice thickness of 1 mm was utilized. The DICOM image dataset was imported into the Varian Eclipse clinical treatment planning system. Target volumes were defined as individual filled wells. Treatment plans were designed to deliver known amounts of radiation dose to the target volumes using the AcurosXB 15.6.06 dose calculation algorithm and a 1 mm dose grid. Mobius3D software, which utilizes an independent beam model and dose calculation algorithm, was used to perform an independent dose calculation to verify the accuracy of the treatment planning system dosimetry. On the day of treatment delivery, a clinical daily quality assurance routine was performed using a Sun Nuclear Daily QA 3 device to verify dose output, and Varian’s integrated machine performance check to verify imaging, mechanical, and a secondary dose output verification. The planned ASTEROIDS setup was replicated on the TrueBeam linear accelerator’s 6 degree of freedom treatment table. Onboard cone beam CT imaging allowed for pretreatment visualization and alignment using translational and rotational couch corrections to within 1 mm. ASTEROIDS were irradiated 4 days after cell loading with 8 and 24 Gy, with a separate unirradiated ASTEROIDS device to serve as the 0 Gy control.

#### Metabolomic analysis

Sample processing was based on a protocol by Xu et al.^68^ Briefly, culture media from the different conditions were thawed on ice and centrifuged for 5 min at 1,000 g at 4 °C. The four sample groups (*n* = 4 for each) used were: A = A549 cells, H = EC cells, and P=PBMCs with 0 or 8 Gy of X-rays and collected at 24 h after exposure. An extraction solution consisted of acetonitrile:methanol:formic acid (75%:25%:0.2%). Nine volumes of extraction solution were mixed with 1 volume of media, vortexed, and centrifuged for 10 min at 18,000 g at 4°C. The supernatant was further filtered through a 0.2 μm Cytiva Nanosep MF centrifugal device with a bio-inert membrane. Recovered material was transferred to glass mass spectrometry (MS) vials. A quality control was also constructed from pooled volumes from each sample. One μL of each sample was injected into a Waters UPLC coupled to a time-of-flight Xevo G2S MS instrument and data collected in MSE function. Details are provided in Data S1. Data deconvolution and putative ID matching were conducted with the software Progenesis QI (Data Analytics). Downstream analysis was conducted with the online software MetaboAnalyst 6.0 using normalized data (“normalized to all compounds” method from Progenesis QI). This method aims to minimize the influence of outliers and create a normal distribution. A heatmap was conducted from the top 100 statistically significant spectral features by combining both positive and negative ionization modes. Positive identification of biomarkers was conducted with tandem MS with pure standards and ramping collision energy to a metabolomics standard initiative (MSI) level 1.^69^

### Quantification and statistical analysis

All experiments were carried out at n = 3–6, where n represents the number of independent experiments or the number of technical replicates from at least 3 independent experiments (see figure captions). Results and error bars in this article are presented as mean ± standard error of the mean (s.e.m.). Graph plotting and statistics were performed on GraphPad Prism (Version 10.4.1). Quantification of sample areas, nuclear 53BP1 foci, and CD45-positive cells on membranes was performed using ImageJ software. Spheroid and HULEC-5a nuclear areas were manually delineated using the ‘Freehand’ selection tool after image calibration with a known scale. PBMCs on membranes were quantified from single fluorescence channel images (CD45) by converting to grayscale, applying an intensity threshold for cell segmentation, performing watershed to separate adjacent cells, and using the ‘Analyze Particles’ function with size and circularity parameters set between 50 and 500 μm^2^ and 0.2–1, respectively. Nuclear 53BP1 foci were quantified by selecting nuclei based on DAPI staining and applying the ‘Find Maxima’ function on the 53BP1 channel with a noise tolerance of 30. For spheroid analysis, only cells within 20 cells from the spheroid edge were counted from single-cell spreads prepared by flattening the spheroids between a microscope slide and coverslip. For large datasets, outliers were identified using the ROUT method with Q set to 1%.[16] Statistical analysis between two conditions was performed by a paired or unpaired Student’s *t* test. For multiple comparisons, one-way or two-way ANOVA with Fisher’s LSD or Tuckey post hoc tests were used. *p*-values <0.05 were considered statistically significant.
