# Supplementary material for: A human 3D culture-organ-on-chip platform for investigating the tumor microenvironment response to ionizing radiation
Source: iScience. 2025 Nov 26;29(1):114236. doi: 10.1016/j.isci.2025.114236 (PMC12757608; doi:10.1016/j.isci.2025.114236)
Supplement: Document S1. Figures S1–S5, Tables S1–S3, and Data S1 [file mmc1.pdf]

iScience, Volume *29*

## Supplemental information

# A human 3D culture-organ-on-chip platform for investigating the tumor microenvironment response to ionizing radiation

**Jerome Lacombe, Sean E. Dunn, Marie Layac, Maria Soldevila, Nabhan M. Fakrudin, Brett Duane, Kurt Chen, Matthew W. Barrett, James Helton, Evagelia C. Laiakis, Albert J. Fornace Jr., Shyam Jani, Stephen Sorensen, Shunjiro Funaki, Aidnag Diaz, and Frederic Zenhausern**

**Supplementary figure S1**

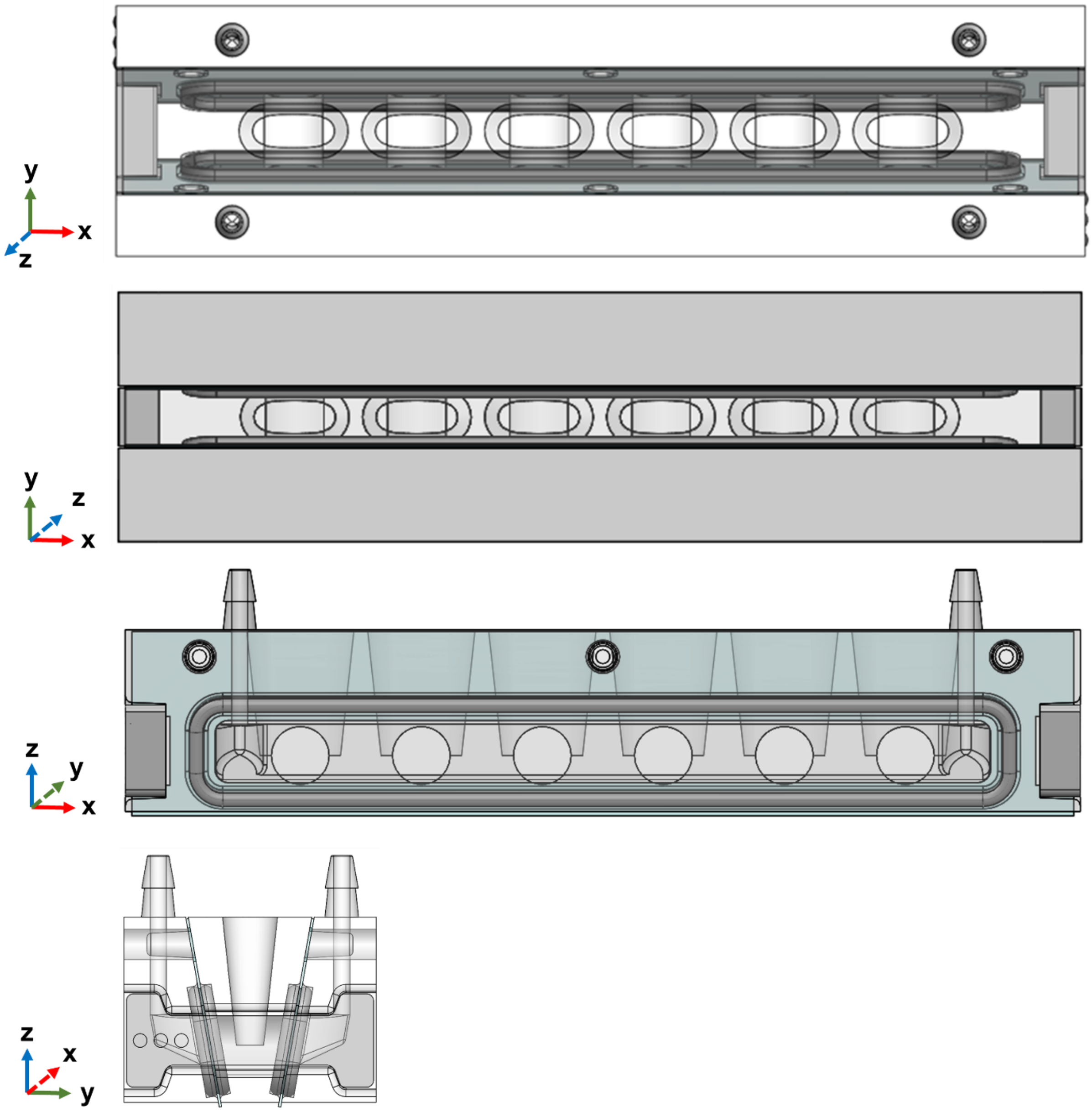


**Figure S1**: **Rotated views of the ASTEROIDS device around the three spatial axes**

**Supplementary figure S2**

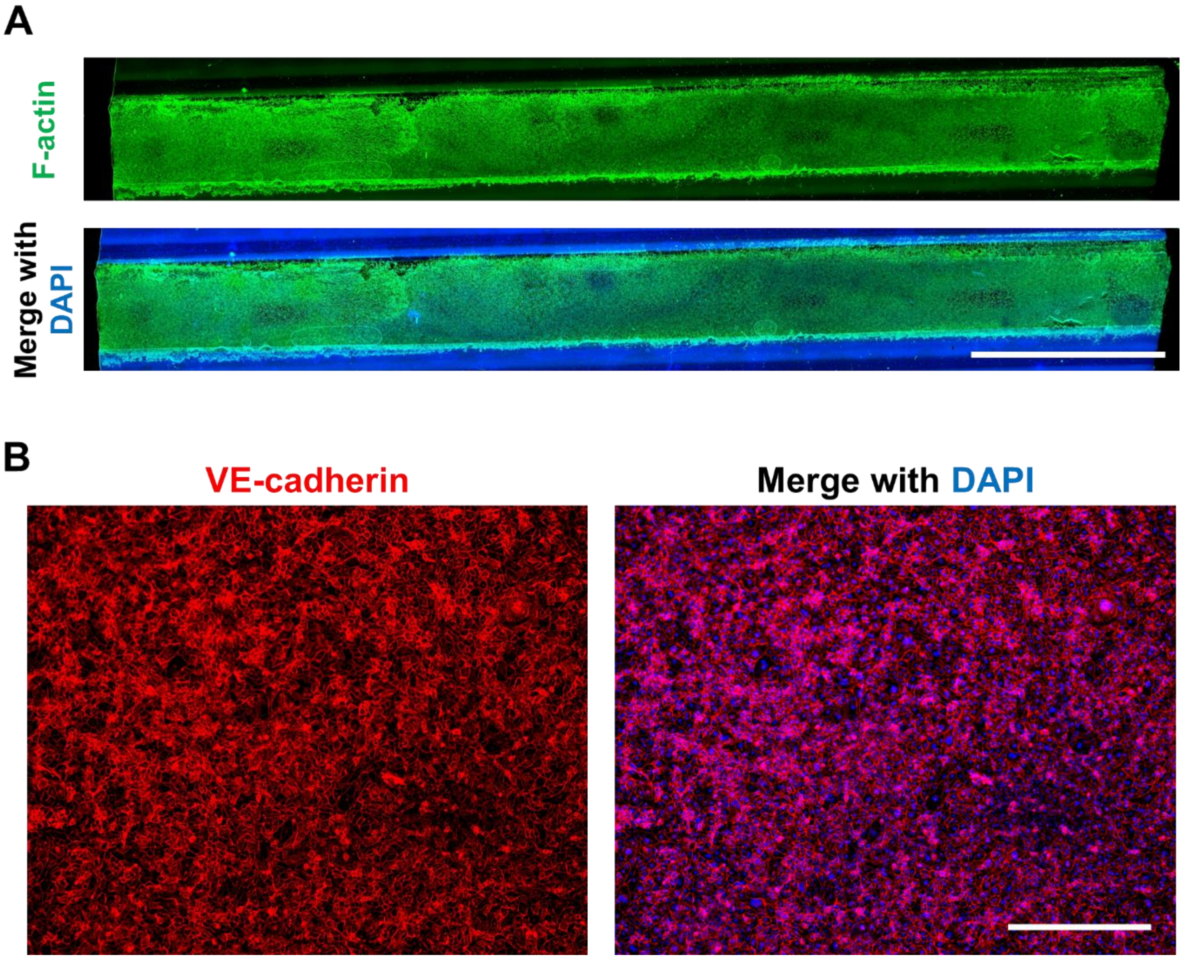


**Figure S2**: **HULEC-5a endothelial barrier within the ASTEROIDS**.
(A) Fluorescent images of the full membrane seeded with HULEC-5a cells, stained for F-actin (green) and counterstained with DAPI (Blue). Scale bar = 1 mm.
(B) Representative fluorescent images of HULEC-5a cells stained for VE-cadherin (red) and counterstained with DAPI (blue). Scale bar = 400 µm.

**Supplementary figure S3**

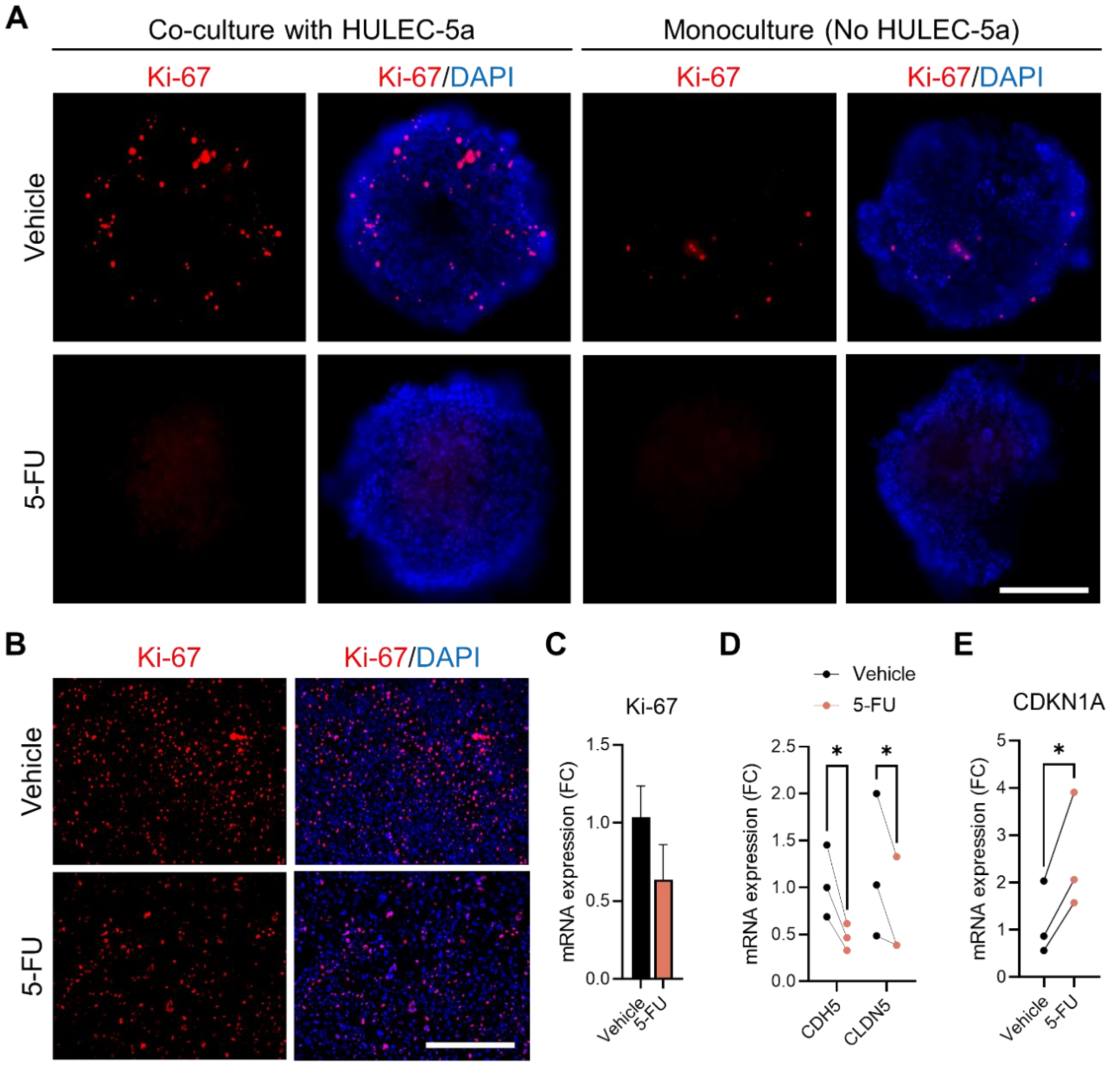


**Figure S3. 5-FU injection in ASTEROIDS vascular compartment affects A549 spheroids and HULEC-5a cells phenotype.**
(A) Fluorescent images of A549 spheroids, cultured in ASTEROIDS in absence or presence of HULEC-5a, 3 days after the injection of DMSO (Vehicle) or 5-FU and immunostained for Ki-67 (red) and counterstained with DAPI (blue). Scale bar = 250 µm.
(B) Fluorescent images of HULEC-5a cells cultured in ASTEROIDS 3 days after injection of DMSO (Vehicle) or 5-FU and immunostained for Ki-67 (red) and counterstained with DAPI (blue). Scale bar = 400 µm.
(C) Ki-67 mRNA level in HULEC-5a cells quantified by qRT-PCR after exposure to DMSO (Vehicle) or 5-FU for 3 days (n = 3).
(D) VE-cadherin (CDH5) and claudin-5 (CLDN5) mRNA level in HULEC-5a cells quantified by qRT-PCR after exposure to DMSO (Vehicle) or 5-FU for 3 days. Differences were calculated using two-way RM ANOVA with Fisher's LSD test (n = 3).
(E) CDKN1A mRNA level in HULEC-5a cells quantified by qRT-PCR after exposure to DMSO (Vehicle) or 5-FU for 3 days. Differences were calculated using paired, two-tailed Student's t-tests with a significance threshold of $\alpha<0.05$ (n = 3).
Data are represented as mean ± s.e.m.

**Supplementary figure S4**

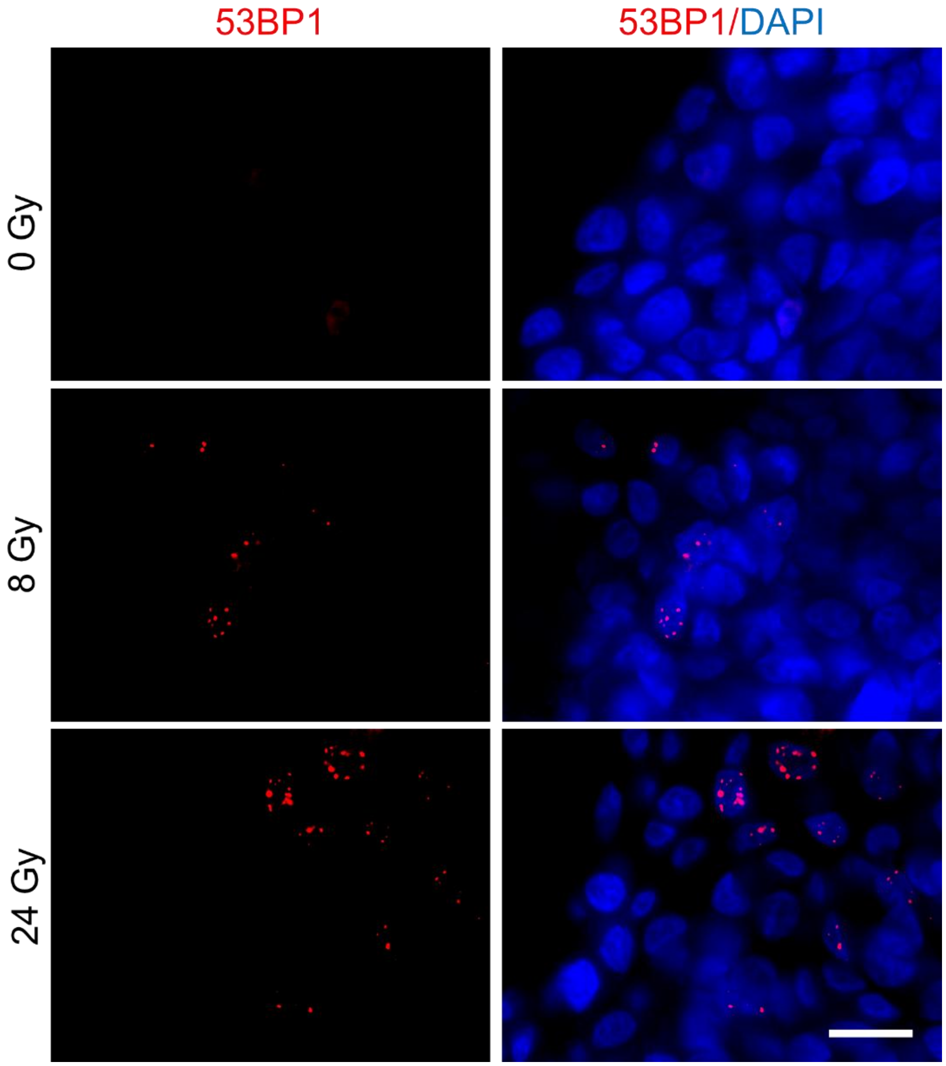


**Figure S4**: Representative fluorescent images of A549 spheroids 24 h after 0, 8 and 24 Gy-irradiation and immunostained for 53BP1. DAPI counterstaining is shown in blue. Scale bar = 50 µm

**Supplementary figure S5**

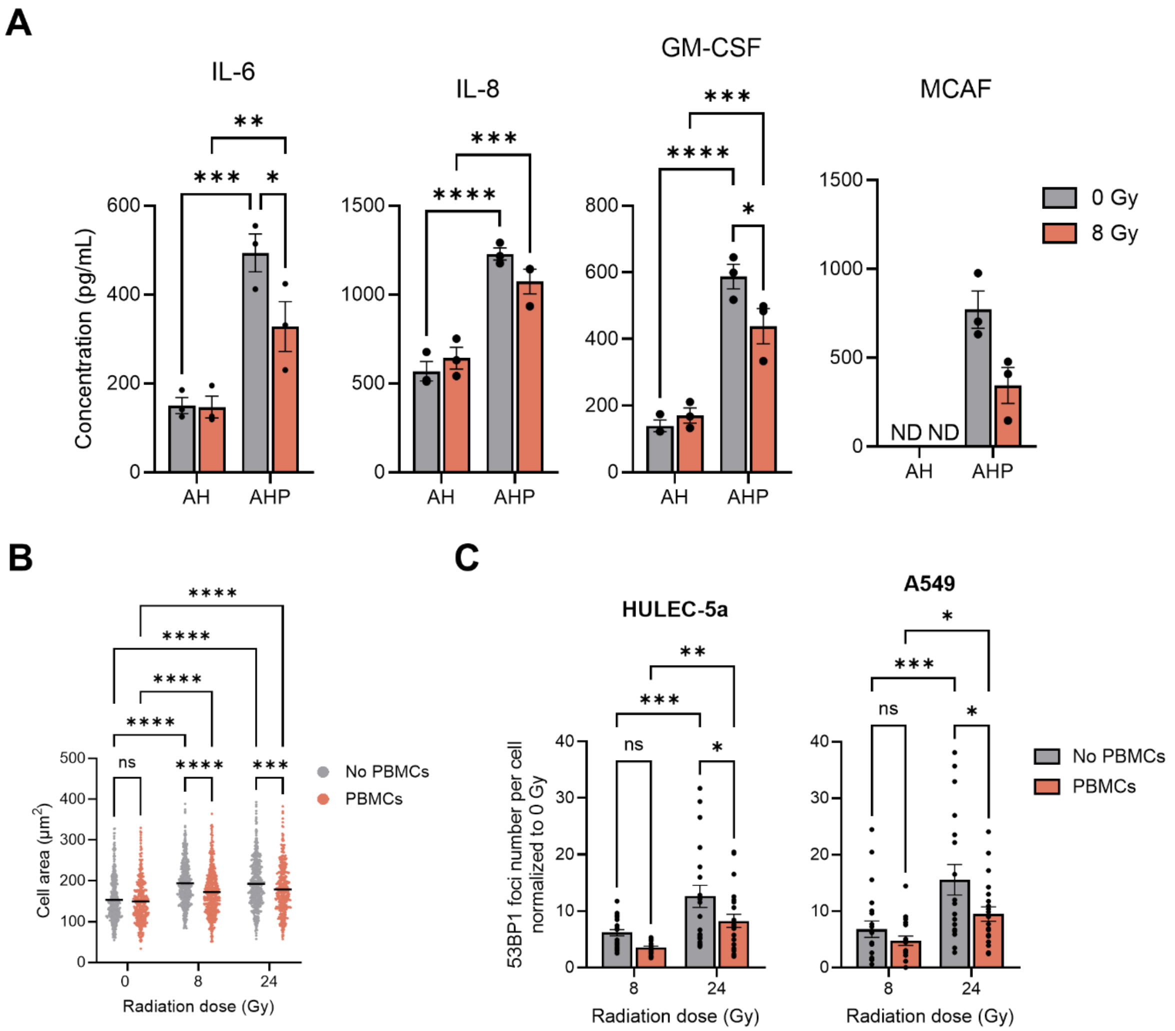


**Figure S5**: **The presence of PBMCs (P) mitigate the effect of irradiation on the co-culture of HULEC-5a cells (H) and A549 spheroids (A) in the ASTEROIDS.**

(A) Expression level of four pro-inflammatory cytokines in ASTEROIDS supernatant cultured for 24 h after 8 Gy-irradiation with HULEC-5a cells + A549 spheroids (AH) or with HULEC-5a cells + A549 spheroids + PBMCs (AHP). Difference was calculated using two-way ANOVA with Fisher's LSD test (n = 3).

(B) Quantification of the nucleus area of HULEC-5a cells cultured with and without PBMCs 24 h after 0, 8 and 24 Gy-irradiation. Differences were calculated using two-way ANOVA with Tukey's multiple comparison test (n > 400 nuclei from 4 independent devices).

(C) Radiation-induced 53BP1 foci per cell normalized to 0 Gy, in HULEC-5a cells and A549 spheroids, cultured with or without PBMCs, 24 h after 8 and 24 Gy-irradiation. Differences were calculated using two-way ANOVA with Fisher's LSD test (n = 17 frames with at least 10 nuclei each from 4 independent devices).

Data are represented as mean ± s.e.m.

**Table S1: Validated metabolites with MS/MS**

| | Validated metabolite | m/z observed | Retention Time | Adduct |
|---|---|---|---|---|
| 1 | Glutamine | 147.0775 | 0.43 | [M+H]+ |
| 2 | Xanthine | 151.0256 | 0.38 | [M-H]- |
| 3 | Pantothenic acid | 218.1028 | 0.73 | [M-H]- |
| 4 | Malic acid | 133.0134 | 0.39 | [M-H]- |
| 5 | Oxoadipic acid | 159.0293 | 0.31 | [M-H]- |
| 6 | Succinic acid | 117.0188 | 0.4 | [M-H]- |
| 7 | Linoleic acid | 279.2342 | 6.85 | [M-H]- |
| 8 | Glutamic acid | 146.0456 | 0.35 | [M-H]- |
| 9 | Taurine | 124.0077 | 0.36 | [M-H]- |

Normalized abundances

| | A+H_0Gy | A+H_0Gy | A+H_0Gy | A+H_0Gy | A+H_8Gy | A+H_8Gy | A+H_8Gy | A+H_8Gy | A+H+P_0Gy | A+H+P_0Gy | A+H+P_0Gy | A+H+P_0Gy | A+H+P_8Gy | A+H+P_8Gy | A+H+P_8Gy | A+H+P_8Gy |
|---|---|---|---|---|---|---|---|---|---|---|---|---|---|---|---|---|
| Glutamine | 109068.5343 | 101555.1852 | 106418.8775 | 94571.10698 | 99924.022 | 100258.079 | 104540.17 | 97105.344 | 100919.9 | 99914.25 | 99331.23 | 93181.55 | 107597.6 | 102844.3 | 95053.46 | 97062.14 |
| Xanthine | 88554.94664 | 101297.577 | 89302.56827 | 86592.83236 | 87474.419 | 94955.1791 | 89436.505 | 93761.275 | 101538.6 | 89072.14 | 88251.75 | 82937.66 | 85976.29 | 102405 | 95183.86 | 83518.58 |
| Pantothenic acid | 1282726.005 | 1259159.095 | 1170745.417 | 1163785.775 | 1318444 | 1188969.21 | 1196870.3 | 1254970.2 | 1233632 | 1137889 | 1194457 | 1091614 | 1213538 | 1178120 | 1172835 | 1097483 |
| Malic acid | 36604.11309 | 40400.85901 | 37393.4629 | 36649.96045 | 37729.018 | 42278.5836 | 37140.018 | 38460.67 | 46196.16 | 46491.46 | 40739.02 | 39754.21 | 42838.29 | 48565.69 | 40448.85 | 37833.65 |
| Oxoadipic acid | 118990.0328 | 126156.3974 | 124886.5536 | 125375.0221 | 120928.61 | 133137.717 | 128958.07 | 124423.92 | 124918.2 | 128921.1 | 134570.5 | 130530.4 | 134067.9 | 131415.9 | 130433.6 | 136328.1 |
| Succinic acid | 129574.5577 | 136543.2601 | 124085.5331 | 117911.1006 | 139573.17 | 135393.745 | 129609.23 | 126704.29 | 127364.9 | 112772.2 | 128208.2 | 117628.1 | 134509 | 121995.3 | 125594.3 | 116737.7 |
| Linoleic acid | 589.1472784 | 2591.137971 | 822.4329651 | 4263.047905 | 10686.372 | 55.9461869 | 1767.6674 | 400.96607 | 182.1884 | 529.189 | 893.4935 | 1427.389 | 0 | 2446.591 | 708.9423 | 2991.901 |
| Glutamic acid | 140496.0381 | 158169.572 | 135241.5289 | 136049.132 | 114297.39 | 142635.098 | 147049.62 | 130373.68 | 147472.7 | 160220 | 161134.7 | 143359.8 | 136642.6 | 157609.7 | 156450.4 | 149991.4 |
| Taurine | 6428.624092 | 7645.220963 | 8909.770432 | 5613.777268 | 8245.3003 | 6960.72758 | 8071.8513 | 6463.2973 | 8602.826 | 14658.34 | 8097.997 | 8588.559 | 10906.11 | 11089.05 | 8969.192 | 9738.068 |

**Table S2: Putative ID's based on HMBD LC-MS database matching and confirmation with Progenesis QI fragmentation patterns**

| | | m/z | Ret time | Metabolic class | Adduct | Putative ID | Error |
|---|---|---|---|---|---|---|---|
| 1 | LipidBlast | 263.2367 | 6.59 | Fatty acids | M+H-H2O | Linoleic acid | 3ppm |
| 2 | | 245.2263 | 6.59 | | | Not a biologically relevant putative match | |
| 3 | no matching | 355.177 | 6.59 | Amide, biotin metabolism intermediate | M+H-H2O | Biocytin | 10ppm |
| 4 | LipidBlast | 321.2402 | 6.59 | Fatty acids | M+H | 18-HETE | 7ppm |
| 5 | no matching | 197.115 | 3.18 | Purine derivative | M+NH4 | N2,N2-dimethylguanine | 2ppm |
| 6 | no matching | 313.7989 | 4.46 | Acylcarnitine | M+H | 3-Oxotridecanoylcarnitine | 7ppm |
| 7 | no matching | 356.1691 | 6.57 | Purine nucleoside | M+NH4 | N-[[(2S,3S,4R,5R)-5-(6-Aminopurin-9-yl)-3,4-dihydroxyoxolan-2-yl]-hydroxymethyl]-N-ethylformamide | 4ppm |
| 8 | no matching | 297.3151 | 6.58 | Long chain fatty alcohols | M-H | Arachidyl alcohol | 4ppm |
| 9 | LipidBlast | 365.2301 | 6.58 | Prostaglandin | M-H | Prostaglandin E2 methyl ester | 9ppm |

**Table S3: Sequence of qRT-PCR primers**

| Gene | Forward primer | Reverse primer |
|---|---|---|
| ANKRD1 | AGAACTGTGCTGGGAAGACG | GCCATGCCTTCAAAATGCCA |
| BBC3 | ACCTCAACGCACAGTACGAG | CCCATGATGAGATTGTACAGGA |
| BAD | GAGTGAGCAGGAAGACTCCAGC | TCCACAAACTCGTCACTCATCC |
| BAX | TGCTTCAGGGTTTCATCCAG | GGCGGCAATCATCCTCTG |
| CCL2 | AGAATCACCAGCAGCAAGTGTCC | TCCTGAACCCACTTCTGCTTGG |
| CDKN1A | AGACCAGCATGACAGATTTCTACC | CTTCCTGTGGGCGGATTAGG |
| COL1A1 | TCTGCGACAACGGCAAGGTG | GACGCCGGTGGTTTCTTGGT |
| COL4A1 | CCAAGGGCGACAGAGGTTTG | ATAAAACTCACCAGGCTCCCC |
| COL6A1 | GAAGAATGTCACCGCCCAGA | GGTGGTGTCAAAGTTGTGGC |
| CLDN1 | GTCTTTGACTCCTTGCTGAATCTG | CACCTCATCGTCTTCCAAGCAC |
| CSF2 | GGAGCATGTGAATGCCATCCAG | CTGGAGGTCAAACATTTCTGAGAT |
| CTGF | AGGAGTGGGTGTGTGACGA | CCAGGCAGTTGGCTCTAATC |
| CTNNB1 | CTGAGGAGCAGCTTCAGTCC | GGCCATGTCCAACTCCATCA |
| CXCL2 | GGCAGAAAGCTTGTCTCAACCC | CTCCTTCAGGAACAGCCACCAA |
| CYR61 | AGCCTCGCATCCTATACAACC | TTCTTTCACAAGGCGGCACTC |
| DDB2 | AGCATCACTGGGCTGAAGTT | TGGTGTCTGAGCTGGCAAAA |
| FN1 | TGAAGAAGGAAAATGGGTGCTT | ACCTCTCCCAACTGGACACT |
| GAPDH | CTCCTGCACCACCAACTGCT | GGGCCATCCACAGTCTTCTG |
| HPRT1 | TGACACTGGCAAAACAATGCA | GGTCCTTTTCACCAGCAAGCT |
| ICAM1 | GCCGGCCAGCTTATACACAA | CAATCCCTCTCGTCCAGTCG |
| IL-6 | CCACCGGGAACGAAAGAGAA | GAGAAGGCAACTGGACCGAA |
| IL-8 | ACTCCAAACCTTTCCACCCC | TTCTCAGCCCTCTTCAAAAACT |
| ITGA1 | TGAAGAAGGAAAATGGGTGCTT | ACCTCTCCCAACTGGACACT |
| ITGB1 | GAGGAGGATTACTTCGGACTTCAG | GCTGGTGTTGTGCTAATGTAAGG |
| ITGB2 | TGCGTCCTCTCTCAGGAGTG | GGTCCATGATGTCGTCAGCC |
| Ki67 | CGTCCCAGTGGAAGAGTTGT | CGACCCCGCTCCTTTTGATA |
| LAMC1 | TCGTCAACGCCGCCTTCAA | GTGTCGGCCTGGTTGTTGTA |
| LAMA5 | CAACGAGTGCCAGTCCTGTA | CACCCTGATAGGTGCCATCC |
| MCAF | AGAATCACCAGCAGCAAGTGTCC | TCCTGAACCCACTTCTGCTTGG |
| OCLN | TCAGGGAATATCCACCTATCACTTCAG | CATCAGCAGCAGCCATGTACTCTTCAC |
| PCNA | CAAGTAATGTCGATAAAGAGGAGG | GTGTCACCGTTGAAGAGAGTGG |
| TAZ | GGACCAAGTACATGAACCACC | TGCAGGACTGGTGATTGGAC |
| VCAM1 | CAAAGGCAGAGTACGCAAACAC | GCTGACCAAGACGGTTGTATCTC |
| VCL | TCAGATGAGGTGACTCGGTTGG | GGGTGCTTATGGTTGGGATTCG |
| VEGFR1 | CCTGCAAGATTCAGGCACCTATG | GTTTCGCAGGAGGTATGGTGCT |
| VEGFR2 | GGAACCTCACTATCCGCAGAGT | CCAAGTTCGTCTTTTCCTGGGC |
| YAP1 | CCCTCGTTTTGCCATGAACC | GTTGCTGCTGGTTGGAGTTG |

**Supplemental datafile 1**

Acquisition Experiment Report

Header
Acquired Date:        21-Dec-2024
Acquired Time:        15:20:16
Task Code:
User Name:
Laboratory Name:
Instrument:           XEVO-G2SQTOF#YDA384K
Conditions:
Submitter:
SampleID:
Bottle Number:        1:A,7
Description:          3

Instrument Calibration:
Calibration File:
Parameters
MS1 Static:       None
MS1 Scanning:
Mass:             50 Da to 1200 Da.
Resolution:       0.0/0.0
Ion Energy:       0.0
Reference File:   ESI_NaFormate_Neg
Acquisition File: Metabolite ID-2024-12-21-08-36-2Ñˆs2
MS1 Scan Speed Compensation:   None
Calibration Time: 08:43
Calibration Date: 12/21/24
Coefficients
MS1 Static:       None
Function 1:        0.000000000120*x^5 + -0.000000016050*x^4 + 0.000000826097*x^3 + -0.000020166389*x^2 + 1.000384214964*x +- 0.004659221084, Root Mass
Function 2:        0.000000000120*x^5 + -0.000000016050*x^4 + 0.000000826097*x^3 + -0.000020166389*x^2 + 1.000384214964*x +- 0.004659221084, Root Mass
Function 3:        0.000000000120*x^5 + -0.000000016050*x^4 + 0.000000826097*x^3 + -0.000020166389*x^2 + 1.000384214964*x +- 0.004659221084, Root Mass

Parameters for C:\Projects\2023.PRO\ACQUDB\13_MSE_NEG.EXP
Created by Masslynx v4.1

Lock Spray Configuration:
Reference Scan Frequency(sec)          10.000
Reference Cone Voltage(V)              30.000
Reference Collision Energy             6.000
Reference DRE Setting                  33.400

Temperature Correction:
Temperature Correction                 Disabled

Instrument Configuration:

| | |
|---|---|
| Lteff | 1800.0 |
| Veff | 6336.90 |
| Resolution | 22000 |
| Min Points in Peak | 2 |
| Acquisition Device | WatersADC |
| ADC Trigger Threshold (V) | -1.00 |
| ADC Input Offset (V) | -1.50 |
| Average Single Ion Intensity | 30 |
| ADC Amplitude Threshold | 2 |
| ADC Centroid Threshold | -1 |
| ADC Ion Area Threshold | 4 |
| ADC Ion Area Offset | 15 |
| ADC Pushes Per IMS Increment | 1 |
| TargetEnhancement Delay Coefficient | 2.1500 |
| TargetEnhancement Delay Offset | 0.0000 |

Experimental Instrument Parameters

Instrument Parameter Filename

C:\Projects\2023.PRO\ACQUDB\G2S.IPR (MODIFIED)

| | |
|---|---|
| Polarity | ES- |
| Analyser | Sensitivity Mode |
| Capillary (kV) | 2.0000 |
| Sampling Cone | 30.0000 |
| Source Temperature (∞C) | 120 |
| Source Offset | 80 |
| Desolvation Temperature (∞C) | 500 |
| Cone Gas Flow (L/Hr) | 25.0 |
| Desolvation Gas Flow (L/Hr) | 1000.0 |
| LM Resolution | 4.7 |
| HM Resolution | 15.0 |
| Aperture 1 | 0.0 |
| Pre-filter | 2.0 |
| Ion Energy | 0.2 |
| Manual Collision Energy | FALSE |
| Collision Energy | 6.0 |
| Detector | 2350 |
| DetectorCache | 0 |
| Sample Infusion Flow Rate (µL/min) | 20 |
| Sample Flow State | LC |
| Sample Fill Volume (µL) | 250 |
| Sample Reservoir | C |
| LockSpray Infusion Flow Rate (µL/min) | 20 |
| LockSpray Flow State | Infusion |
| LockSpray Reservoir | B |
| LockSpray Capillary (kV) | 1.00 |
| Use Manual LockSpray Collision Energy | FALSE |
| Collision Energy | 6.0 |
| Acceleration1 | 10.0 |
| Acceleration2 | 80.0 |
| Aperture2 | 15.0 |
| Transport1 | 30.0 |
| Transport2 | 30.0 |
| Steering | 0.00 |

| | |
|---|---|
| Tube Lens | 15 |
| Pusher | 1900.0 |
| Pusher Offset | -0.33 |
| Puller | 1400.0 |
| Pusher Cycle Time (µs) | Automatic |
| Pusher Width (µs) | Automatic |
| Collector | 60 |
| Collector Pulse | 10.0 |
| Stopper | 10 |
| Stopper Pulse | 20.0 |
| Entrance | 13 |
| Static Offset | 120 |
| Puller Offset | 0.00 |
| Reflectron Grid (kV) | 1.704 |
| Flight Tube (kV) | 9.00 |
| Reflectron (kV) | 1.602 |
| TWave Entrance | 2.00 |
| Static Offset | 120.00 |
| TWave Exit | 15.00 |
| TWave Trap Height | 4.00 |
| TWave Extract Height | 15.00 |
| Trap Wave Velocity (m/s) | 260 |
| Trap Wave Height (V) | 0.2 |
| Step Wave 1 In Manual Control | OFF |
| Enable Reverse Operation | OFF |
| Step Wave 1 In Velocity (m/s) | 300.0 |
| Step Wave 1 In Height | 15.0 |
| Step Wave 1 Out Manual Control | OFF |
| Step Wave 1 Out Velocity (m/s) | 300.0 |
| Step Wave 1 Out Height | 15.0 |
| Step Wave 2 Manual Control | OFF |
| Step Wave 2 Velocity (m/s) | 300.0 |
| Step Wave 2 Height | 1.0 |
| Use Manual Step Wave DC | OFF |
| Step Wave TransferOffset | 25.0 |
| Step Wave DiffAperture1 | 3.0 |
| Step Wave DiffAperture2 | 0.0 |
| Use Automatic RF Settings | TRUE |
| StepWave1RFOffset | 300.0 |
| StepWave2RFOffset | 350.0 |
| Backing | 3.70e0 |
| Collision | 1.18e-2 |
| TOF | 5.07e-7 |
| TrapRFOffset | 380 |
| MS Profile Type | Auto P |
| MSProfileMass1 | 100 |
| MSProfileDwellTime1 | 20 |
| MSProfileRampTime1 | 20 |
| MSProfileMass2 | 300 |
| MSProfileDwellTime2 | 20 |
| MSProfileRampTime2 | 40 |
| MSProfileMass3 | 500 |
| Use Automatic RF Settings | TRUE |
| AutoStepWave1RFOffset | 300 |

```
AutoStepWave2RFOffset                      350
LockMassValidSigma                         5

Acquisition mass range
Start mass                        50.000
End mass                          1200.000
Calibration mass range
Start mass                        0.000
End mass                          0.000

Experiment Reference Compound Name: Lockspray MS

Function Parameters - Function 1 - TOF PARENT FUNCTION
[ACQUISITION]
Survey Start Time                 0.0
Survey End Time                            13.0
Survey Ion Mode                            ES Mode
Survey Polarity                            Negative
[PARENT MS SURVEY]
Survey Start Mass                 50.0
Survey End Mass                            1200.0
Parent Survey High CE (V)                  30.0
Parent Survey Low CE (V)                   10.0
TIC Threshold                              5.0
Survey Scan Time                  0.2
Survey Interscan Time                      0.0
Survey Data Format                         Centroid
ADC Sample Frequency (GHz)                 6.0
ADC Pusher Frequency (µs)                  60.0
ADC Pusher Width (µs)                      1.50
Survey Use Tune Page CV                          YES
[PRODUCT IONS]
Use High CE Product Ions Mass List File          NO
High CE Product Ions Mass List Filename
Product Ions Match Logic                   NO
Product Ions Switch Threshold (Intensity/s)   10.0
Product Ions Switch Detection Window +/- (mDa)      100.0
Product Ions Retention Time Window +/- (sec)  10.0
[NEUTRAL LOSS]
Use Neutral Loss Mass List File            NO
Neutral Loss Mass List Filename
Neutral Loss Match Logic                   OR
Neutral Loss Switch Threshold (Intensity/s)   10.0
Neutral Loss Switch Detection Window +/- (mDa)      100.0
[MS/MS]
MSMS Start Mass                            50.0
MSMS End Mass                              1200.0
Number of components                       0
Use MSMS to MS Switch After Time           NO
MSMS Switch After Time (sec)               10.0
Absence of Neutral Loss                          NO
Absence of Product Ion                     NO
MSMS Scan Time (sec)                       1.0
MSMS Interscan Time (sec)                  0.0
```

| | |
|---|---|
| MSMS Data Format | Continuum |
| Use Tune Page Cone Voltage | YES |
| Use MS/MS ipr File | NO |
| Instrument Parameter Filename | |
| [PEAK DETECTION] | |
| Peak Detection Window | 1.0 |
| Use Intensity based Peak Detection | YES |
| Charge State Tolerance Window | 3.0 |
| Charge State Extraction Window | 4.0 |
| Deisotope Tolerance Window | 3.0 |
| Deisotope Extraction Window | 4.0 |
| Discard survey data | NO |
| [COLLISION ENERGY] | |
| Using Auto MS Collision Energy (eV) | 6.000000 |
| [INCLUDE] | |
| Precursor Selection | Everything |
| [EXCLUDE] | |
| Use Exclude Masses List | NO |
| Exclude Mass Range | |
| Use Exclude File Masses | NO |
| Exclude Mass Filename | |
| Exclude Window +/- (mDa) | 100.0 |
| Exclude Retention Time Window | 10.0 |
| Reference Centroid Average | 0 |
| Reference Frequency | 0.0 |
| Reference Cone Voltage | 0.0 |
| Dynamic Range | Extended |
| Calibration | Dynamic 2 |

Function Parameters - Function 2 - TOF PARENT FUNCTION

| | |
|---|---|
| [ACQUISITION] | |
| Survey Start Time | 0.0 |
| Survey End Time | 13.0 |
| Survey Ion Mode | ES Mode |
| Survey Polarity | Negative |
| [PARENT MS SURVEY] | |
| Survey Start Mass | 50.0 |
| Survey End Mass | 1200.0 |
| Ramp High Energy from | 10.0 to 50.0 |
| Parent Survey Low CE (V) | 10.0 |
| TIC Threshold | 5.0 |
| Survey Scan Time | 0.2 |
| Survey Interscan Time | 0.0 |
| Survey Data Format | Centroid |
| ADC Sample Frequency (GHz) | 6.0 |
| ADC Pusher Frequency (µs) | 60.0 |
| ADC Pusher Width (µs) | 1.50 |
| Survey Use Tune Page CV | YES |
| [PRODUCT IONS] | |
| Use High CE Product Ions Mass List File | NO |
| High CE Product Ions Mass List Filename | |
| Product Ions Match Logic | NO |
| Product Ions Switch Threshold (Intensity/s) | 10.0 |
| Product Ions Switch Detection Window +/- (mDa) | 100.0 |

```
Product Ions Retention Time Window +/- (sec)  10.0
[NEUTRAL LOSS]
Use Neutral Loss Mass List File         NO
Neutral Loss Mass List Filename
Neutral Loss Match Logic                OR
Neutral Loss Switch Threshold (Intensity/s)   10.0
Neutral Loss Switch Detection Window +/- (mDa)      100.0
[MS/MS]
MSMS Start Mass                         50.0
MSMS End Mass                           1200.0
Number of components                    0
Use MSMS to MS Switch After Time        NO
MSMS Switch After Time (sec)            10.0
Absence of Neutral Loss                       NO
Absence of Product Ion                  NO
MSMS Scan Time (sec)                    1.0
MSMS Interscan Time (sec)               0.0
MSMS Data Format                  Continuum
Use Tune Page Cone Voltage              YES
Use MS/MS ipr File                      NO
Instrument Parameter Filename
[PEAK DETECTION]
Peak Detection Window                   1.0
Use Intensity based Peak Detection      YES
Charge State Tolerance Window                 3.0
Charge State Extraction Window                4.0
Deisotope Tolerance Window              3.0
Deisotope Extraction Window             4.0
Discard survey data                     NO
[COLLISION ENERGY]
MS Collision Energy Low (eV)            10.0
MS Collision Energy High (eV)                 50.0
[INCLUDE]
Precursor Selection                     Everything
[EXCLUDE]
Use Exclude Masses List                       NO
Exclude Mass Range
Use Exclude File Masses                       NO
Exclude Mass Filename
Exclude Window +/- (mDa)                100.0
Exclude Retention Time Window                 10.0
Reference Centroid Average              0
Reference Frequency                     0.0
Reference Cone Voltage                  0.0
[EXPRESSION]
Collision Energy Ramp Start (eV)        10.0
Collision Energy Ramp End (eV)                50.0
Calibration                       Dynamic 2

Function Parameters - Function 3 - TOF PARENT FUNCTION
[ACQUISITION]
Survey Start Time                 0.0
Survey End Time                         13.0
Survey Ion Mode                         ES Mode
```

```
Survey Polarity                         Negative
[PARENT MS SURVEY]
Survey Start Mass                  50.0
Survey End Mass                         1200.0
Parent Survey High CE (V)               30.0
Parent Survey Low CE (V)                10.0
TIC Threshold                           5.0
Survey Scan Time                   0.3
Survey Interscan Time                   0.1
Survey Data Format                      Centroid
ADC Sample Frequency (GHz)              6.0
ADC Pusher Frequency (μs)               60.0
ADC Pusher Width (μs)                   1.50
Survey Use Tune Page CV                      YES
[PRODUCT IONS]
Use High CE Product Ions Mass List File      NO
High CE Product Ions Mass List Filename
Product Ions Match Logic                NO
Product Ions Switch Threshold (Intensity/s)  10.0
Product Ions Switch Detection Window +/- (mDa)     100.0
Product Ions Retention Time Window +/- (sec)  10.0
[NEUTRAL LOSS]
Use Neutral Loss Mass List File         NO
Neutral Loss Mass List Filename
Neutral Loss Match Logic                OR
Neutral Loss Switch Threshold (Intensity/s)  10.0
Neutral Loss Switch Detection Window +/- (mDa)     100.0
[MS/MS]
MSMS Start Mass                         100.0
MSMS End Mass                           1500.0
Number of components                    1
Use MSMS to MS Switch After Time        NO
MSMS Switch After Time (sec)            10.0
Absence of Neutral Loss                      NO
Absence of Product Ion                  NO
MSMS Scan Time (sec)                    1.0
MSMS Interscan Time (sec)               0.1
MSMS Data Format                   Continuum
Use Tune Page Cone Voltage              YES
Use MS/MS ipr File                      NO
Instrument Parameter Filename
[PEAK DETECTION]
Peak Detection Window                   1.0
Use Intensity based Peak Detection      YES
Charge State Tolerance Window                3.0
Charge State Extraction Window               4.0
Deisotope Tolerance Window              3.0
Deisotope Extraction Window             4.0
Discard survey data                     NO
[COLLISION ENERGY]
Using Auto MS Collision Energy (eV)          6.000000
[INCLUDE]
Precursor Selection                     Everything
[EXCLUDE]
```

```
Use Exclude Masses List                     NO
Exclude Mass Range
Use Exclude File Masses                     NO
Exclude Mass Filename
Exclude Window +/- (mDa)               100.0
Exclude Retention Time Window               10.0
Reference Centroid Average             0
Reference Frequency                    0.0
Reference Cone Voltage                 0.0
Dynamic Range                          Extended
Calibration                       Dynamic 2
```

ACE Experimental Record

Inlet Method File:
c:\projects\2023.pro\acqudb\behc18_celltissueextract_40c_soldwash_abc

--------------------- Run method parameters ----------------

-- PUMP --

```
Waters ACQUITY QSM
 Solvent A Name: Water+0.1%FA
 Solvent B Name: Acetonitrile+0.1%FA
 Solvent C Name: Isopropanol+0.1%FA
 Solvent D Name:
 Low Pressure Limit: 0 psi
 High Pressure Limit: 15000 psi
 Seal Wash Period: 20.00 min
 [Gradient Table]
  Time(min) Flow Rate(mL/min) %A %B %C %D Curve
 1. Initial 0.400 95.0 5.0 0.0 0.0 Initial
 2. 0.50 0.400 95.0 5.0 0.0 0.0 6
 3. 8.00 0.400 2.0 98.0 0.0 0.0 6
 4. 9.00 0.400 0.0 11.8 88.2 0.0 6
 5. 10.50 0.400 0.0 11.8 88.2 0.0 6
 6. 11.50 0.400 50.0 50.0 0.0 0.0 6
 7. 12.50 0.400 95.0 5.0 0.0 0.0 6
 8. 13.00 0.400 95.0 5.0 0.0 0.0 6
 Comment: ABC
 Flow Ramp Rate: 0.45 min
 D Solvent Selection (if supported): No Change
 System Pressure Data Channel: Yes
 Flow Rate Data Channel: No
 %A Data Channel: Yes
 %B Data Channel: Yes
 %C Data Channel: Yes
 %D Data Channel: No
 Primary Data Channel: No
 Accumulator Data Channel: No
 Degasser Data Channel: No
```

Gradient Start: At Injection
Gradient Start Volume: 0 uL
Gradient Start Time: 0.00 min
Participate in pre-analysis: No

-- END PUMP --

-- DETECTOR --

Waters Acquity CM

Target Column Temperature: 40.0 C
Temperature Alarm Band: 5.0 C
Shutdown all columns: No
Column Valve Position: Column 2
Equilibration Time: 0.5 min
Active Preheater: Use Console Configuration
External Valve 1: Position 1
External Valve 2: No Change
External Valve 3: No Change
Comment: BEH C18 50 mm
Column Temperature Data Channel: No
Preheater Temperature Data Channel: No

-- END DETECTOR --

-- AUTOSAMPLER --

Waters ACQUITY FTN AutoSampler

Sample Run Injection Parameter

Injection Volume (ul) - 1.00
-- END AUTOSAMPLER --

---------------------------- oOo -----------------------------

End of experimental record.

------------------- Waters ACQUITY QSM Postrun Report ---------------

Firmware Version: 1.65.287 (Apr 17 2015

Software Version: 1.69.2400

Checksum: 0xaf834c27

Serial Number: D14QSM424A

Minimum System Pressure: 2355.0 psi

Maximum System Pressure: 7416.0 psi

Average System Pressure: 4694.0 psi

---------------------------- oOo -----------------------------

------------------- Waters ACQUITY FTN Postrun Report ---------------

Software Version: 1.69.2261

Firmware Version: 1.65.375 (Mar 26 2015)

Checksum: 0x34728d7d

Serial Number: D14SDI239G

Sample Syringe Size: 100.0

Extension Loop Size: 0.0

Needle Size: 15.0

Minimum Sample Temperature: 5.9 C

Maximum Sample Temperature: 6.1 C

Average Sample Temperature: 6.0 C

Minimum Column Temperature: -0.2 C

Maximum Column Temperature: 0.0 C

Average Column Temperature: -0.2 C

---------------------------- oOo -----------------------------

------------------- Waters Acquity CM Postrun Report ---------------

Software Version: 1.69.2942
Firmware Version: 1.69.154 (Feb 17 2017)

Checksum: 0x17ac4a7

Serial Number: E14CMP465G

Valve Position: 2

ColumnType: ACQUITY UPLCÆ BEH C18 1.7µm

Column Serial Number: 046533193351

Column Part Number: 186002350

Total Injections on Column: 2702

Minimum Column Temperature: 40.0 C

Maximum Column Temperature: 40.0 C

Average Column Temperature: 40.0 C

---------------------------- oOo -----------------------------

------------------- Active eCord Data ---------------

Valve Current Position: 2
Column Name: ACQUITY UPLCÆ BEH C18 1.7µm
Part Number: 186002350
Serial Number: 046533193351
Injection Count: 2702
Injection Count Threshold: 0
Date of First Injection: 11/6/2024 8:42:7 AM
Date of Last Injection: 12/21/2024 3:20:42 PM
Sample Count: 0
Sample Set Count: 25
Maximum Pressure: 7430 psi
Date of Maximum Pressure: 12/21/2024 1:25:4 PM
Maximum Temperature: 60.2 ∞C
Date of Maximum Temperature: 11/6/2024 1:52:48 PM

---------------------------- oOo -----------------------------

Function 1
Scans in function: 864
Cycle time (secs): 0.214
Scan duration (secs): 0.200

Inter Scan Delay (secs): 0.014
Start and End Time(mins): 0.000 to 13.000
Ionization mode: ES-
Data type: Enhanced Accurate Mass
Function type: TOF MS
Mass range: 50 to 1200

Function 2
Scans in function: 864
Cycle time (secs): 0.214
Scan duration (secs): 0.200
Inter Scan Delay (secs): 0.014
Start and End Time(mins): 0.000 to 13.000
Ionization mode: ES-
Data type: Enhanced Accurate Mass
Function type: TOF MS
Mass range: 50 to 1200

Function 3
Scans in function: 76
Cycle time (secs): 0.400
Scan duration (secs): 0.300
Inter Scan Delay (secs): 0.100
Start and End Time(mins): 0.000 to 13.000
Ionization mode: ES-
Data type: Enhanced Accurate Mass
Function type: TOF MS
Mass range: 50 to 1200
